# Protein Deimination and Extracellular Vesicle Profiles in Antarctic Seabirds

**DOI:** 10.3390/biology9010015

**Published:** 2020-01-08

**Authors:** Richard A. Phillips, Igor Kraev, Sigrun Lange

**Affiliations:** 1 British Antarctic Survey, Natural Environment Research Council, Cambridge CB3 0ET, UK; raphil@bas.ac.uk; 2 Electron Microscopy Suite, Faculty of Science, Technology, Engineering and Mathematics, Open University, Milton Keynes MK7 6AA, UK; igor.kraev@open.ac.uk; 3 Tissue Architecture and Regeneration Research Group, School of Life Sciences, University of Westminster, London W1W 6UW, UK

**Keywords:** Peptidylarginine deiminases (PADs), protein deimination, extracellular vesicles (EVs), Antarctic seabirds (wandering albatross (*Diomedea exulans*), grey-headed albatross (*Thalassarche chrysostoma*), black-browed albatross (*Thalassarche melanophris*), northern giant petrel (*Macronectes halli*), southern giant petrel (*Macronectes giganteus*), white-chinned petrel (*Procellaria aequinoctialis*), brown skua (*Stercorarius antarcticus*), south polar skua (*Stercorarius maccormicki*)), immunity, metabolism

## Abstract

Pelagic seabirds are amongst the most threatened of all avian groups. They face a range of immunological challenges which seem destined to increase due to environmental changes in their breeding and foraging habitats, affecting prey resources and exposure to pollution and pathogens. Therefore, the identification of biomarkers for the assessment of their health status is of considerable importance. Peptidylarginine deiminases (PADs) post-translationally convert arginine into citrulline in target proteins in an irreversible manner. PAD-mediated deimination can cause structural and functional changes in target proteins, allowing for protein moonlighting in physiological and pathophysiological processes. PADs furthermore contribute to the release of extracellular vesicles (EVs), which play important roles in cellular communication. In the present study, post-translationally deiminated protein and EV profiles of plasma were assessed in eight seabird species from the Antarctic, representing two avian orders: Procellariiformes (albatrosses and petrels) and Charadriiformes (waders, auks, gulls and skuas). We report some differences between the species assessed, with the narrowest EV profiles of 50–200 nm in the northern giant petrel *Macronectes halli*, and the highest abundance of larger 250–500 nm EVs in the brown skua *Stercorarius antarcticus*. The seabird EVs were positive for phylogenetically conserved EV markers and showed characteristic EV morphology. Post-translational deimination was identified in a range of key plasma proteins critical for immune response and metabolic pathways in three of the bird species under study; the wandering albatross *Diomedea exulans*, south polar skua *Stercorarius maccormicki* and northern giant petrel. Some differences in Gene Ontology (GO) biological and Kyoto Encyclopedia of Genes and Genomes (KEGG) pathways for deiminated proteins were observed between these three species. This indicates that target proteins for deimination may differ, potentially contributing to a range of physiological functions relating to metabolism and immune response, as well as to key defence mechanisms. PAD protein homologues were identified in the seabird plasma by Western blotting via cross-reaction with human PAD antibodies, at an expected 75 kDa size. This is the first study to profile EVs and to identify deiminated proteins as putative novel plasma biomarkers in Antarctic seabirds. These biomarkers may be further refined to become useful indicators of physiological and immunological status in seabirds—many of which are globally threatened.

## 1. Introduction

Peptidylarginine deiminases (PADs) are calcium-dependent enzymes which post-translationally convert arginine into citrulline in target proteins in an irreversible manner. This protein deimination can lead to structural and functional changes in target proteins [1,2,3,4]. Structures most prone to deimination are beta-sheets and intrinsically disordered proteins, and identified deiminated targets to date include nuclear, cytoplasmic and mitochondrial proteins [2,4,5,6,7,8,9,10,11]. Protein deimination can affect gene regulation and cause generation of neo-epitopes [5,12] but may also allow for protein moonlighting, facilitating several physiologically relevant functions from within one polypeptide chain [13,14]. PADs have been identified in diverse taxa from bacteria to mammals, with five tissue-specific PAD isozymes in mammals, three in the chicken *Gallus gallus domesticus*, one in bony fish [1,6,7,15,16] and PAD homologues in bacteria, protozoa and fungi [17,18,19,20]. Although PADs are well known to have pathophysiological roles in cancer, autoimmune and central nervous system (CNS) diseases [4,5,12,16,21,22], much less is known about their involvement in physiological processes. Recent comparative animal studies have therefore focused on elucidating roles for post-translational deimination in immunological and metabolic pathways in a wide range of animal species [6,7,8,9,10,11,23,24]. In birds, PADs have been implicated in tissue regeneration of the chicken CNS, including via inflammatory pathways [16], but their roles in physiology and immunology of birds in general remain to be fully understood.

PADs play crucial roles in the cellular release of extracellular vesicles (EVs) in diverse taxa [18,19,25,26,27]. EVs are found in most body fluids and participate in cellular communication via transfer of cargo proteins and genetic material [5,28,29,30,31]. EVs isolated from a range of bodyfluids, including plasma, have been identified as usable biomarkers for assessment of health and can be indicative of pathological processes [32,33]. Hitherto, the main body of EV research has been in the context of human pathologies; however, recent studies assessing EVs in comparative animal models reflect an increasing interest in elucidating roles for EVs throughout the phylogenetic tree [8,9,10,11,19,24,34,35]. Differences in EV profiles among taxonomic groups have indeed been reported in a range of taxa. Human EVs are generally observed in a narrow size range from 30 to 300 nm [36] and similar size-ranges of EV size profiles have been reported in naked mole-rats (*Heterocephalus glaber*) [11]. In teleost fish EVs were reported in higher abundance at 300–500 nm [8,35], while in elasmobranches higher abundance of small EVs in the 10–200 nm size range are reported [9]. In the protozoa *Giardia intestinalis*, two distinct EV size populations with different functions in host-pathogen interactions have been described [19]. In bacteria, EV profiles from Gram-negative and Gram-positive bacteria have been described in the size range of 10–600 nm and 60–400 nm and were also shown to change with respect to size profile and EV cargo in response to drug-treatment [18,37]. In camelids EVs are reported in llama (*Lama glama*) plasma in the 40–400 nm range [10]. In human cancer studies, cellular EV profiles vary between cancer types and change in response to drug-treatment, both with respect to EV size distribution and cargo [25,26,27,37]. A recent study assessing serum EVs from teleost fish (cod, *Gadus morhua* L.), reported changes in EV release and cargo (deiminated proteins and microRNAs) related to immunological status and growth in response to change in water temperature during rearing [38]. Hitherto, no studies on EVs have been carried out in seabirds, despite the potential for assessments of physiological status or the level of environmental or immunological challenges.

Seabirds are subject to a range of natural and anthropogenic pressures, including from incidental mortality (bycatch) in fisheries, overfishing, invasive species and exposure to pathogens and contaminants [39,40,41]. In addition, global climate change affects prey abundance and distribution at sea, increases the frequency of extreme weather (storms, high winds, rainfall or heatwaves) and possibly the likelihood or severity of disease outbreaks [42,43,44]. Numerous studies have examined levels of a range of heavy metal and other contaminants [39,45,46,47]. Similarly, a range of seabird species have been screened for specific pathogens [48], including for the agent of avian cholera (*Pasteurella multocida*) [49,50,51,52], avian pox [53] as well as other bacterial [54], viral [55,56] and parasitic infections [57,58,59,60,61]. However, less research has been carried out on immunological markers, which should be indicative of general health in seabirds [62,63,64,65,66]. This compares with poultry, for example, in which acute-phase proteins have been studied because of commercial interests in minimizing disease outbreaks on farms [67,68,69,70,71]. As seabirds provide an ideal model for assessment of environmental changes and belong to the most globally threatened of all groups of birds [40,41], the identification of novel biomarkers to assess their health status is of pivotal importance.

In the current baseline study, plasma EV profiles were assessed in one individual from eight seabird species representing two avian orders: Procellariiformes (albatrosses and petrels) and Charadriiformes (waders, auks, gulls and skuas). Furthermore, deiminated protein profiles were assessed in plasma of three species. Our findings indicate some differences in EV profiles and reveal that a range of key immune and metabolic proteins are post-translationally deiminated in plasma of seabirds. Our findings further current understanding of moonlighting functions of such proteins both in physiological and pathophysiological processes in birds. In addition, EVs and deimination profiles have potential value as novel biomarkers to assess immunological and general health status of seabirds.

## 2. Materials and Methods

### 2.1. Sampling of Seabird Plasma

Blood was collected from one adult individual of each of the following eight seabird species during the breeding season: wandering albatross (*Diomedea exulans*), grey-headed albatross (*Thalassarche chrysostoma*), black-browed albatross (*Thalassarche melanophris*), southern giant petrel (*Macronectes giganteus*), northern giant petrel (*Macronectes halli*), white-chinned petrel (*Procellaria aequinoctialis*) and brown skua (*Stercorarius antarcticus*) at Bird Island, South Georgia (54°00′ S, 38°03′ W), and south polar skua (*Stercorarius maccormicki*) at Rothera Point, Adelaide Island (67°04′ S, 68°07′ W). All individuals appeared to be good health at the time of sampling. Sample collection was approved by the British Antarctic Survey Animal Welfare and Ethical Review Committee and conducted under permits from the Government of South Georgia and the South Sandwich Islands, and UK Foreign and Commonwealth Office. Volumes of 1.0–2.0 mL of blood per bird (one bird per species) were collected in lithium heparin paediatric tubes, and plasma was separated by centrifuging at 750× *g* for 10 min. Sampling conditions, procedures and processing were similar in all cases, and should therefore not contribute to sample variation. Plasma was immediately frozen at −20 °C until further use. EVs isolated from the individual bird plasma sample were characterised by size exclusion using nanoparticle tracking analysis (NTA), by Western blotting, using EV-specific protein markers and by morphological analysis using transmission electron microscopy (TEM).

### 2.2. Extracellular Vesicle Isolation and NTA Analysis

Plasma samples from individual birds, were thawed and EVs isolated by step-wise centrifugation according to established protocols using ultracentrifugation and the recommendations of MISEV2018 (the minimal information for studies of extracellular vesicles 2018; [72]). The plasma was diluted 1:4 in ultrafiltered (using a 0.22 μm filter) Dulbecco’s PBS (250 μL plasma added to 750 μL DPBS) and then centrifuged at 4000× *g* for 30 min at 4 °C for removal of aggregates and apoptotic bodies. The supernatant was collected and centrifuged at 100,000× *g* for 1 h at 4 °C. The resulting EV-enriched pellet was resuspended in DPBS, centrifuged again at 100,000× *g* for 1 h at 4 °C and thereafter resuspended in 100 µL DPBS and frozen at −80 °C until further analysis. For nanoparticle tracking analysis (NTA), each EV pellet was diluted 1/100 in DPBS (10 μL EV pellet diluted in 990 μL DPBS) and analysed by NTA, based on Brownian motion of particles in suspension [73], using the NanoSight NS300 system (Malvern Panalytical Ltd., Malvern, UK). The NanoSight system was used in conjunction with a syringe pump to ensure continuous flow of the sample, with approximately 40–60 particles per frame and videos recorded for 5 × 60 s. Replicate histograms generated from the recordings were averaged using the Nanosight NS300 software (Malvern).

### 2.3. Transmission Electron Microscopy (TEM)

The EV pellets obtained from plasma, as described above for each individual, were fixed with 2.5% glutaraldehyde in 100 mM sodium cacodylate buffer (pH 7.0) for 1 h at 4 °C. EVs were then resuspended in 100 mM sodium cacodylate buffer (pH 7.0) and placed on to a grid with a glow-discharged carbon support film. The EVs were stained with 2% aqueous Uranyl Acetate (Sigma-Aldrich, Gillingham, UK) and imaged by using transmission electron microscopy (TEM) with a Morada CCD camera (EMSIS GmbH, Münster, Germany), processed via iTEM (EMSIS).

### 2.4. Western Blotting

For protein analysis, bird plasma and plasma-EVs (each EV preparation derived from 250 µL plasma, reconstituted in 100 µL PBS after isolation and purification as before) were diluted 1:1 in 2× Laemmli sample buffer, boiled for 5 min at 100 °C and separated by SDS-PAGE on 4–20% TGX gels (BioRad, Watford, UK). Following SDS-PAGE, proteins were transferred to nitrocellulose membranes using semi-dry Western blotting. The membranes were blocked in 5% bovine serum albumin (BSA, Sigma-Aldrich, Gillingham, UK) in tris-buffered saline (TBS-T, containing 0.1% Tween-20, BioRad) for 1 h at room temperature (RT) and incubated overnight at 4 °C with the following primary antibodies diluted in TBS-T: F95 (pan-deimination antibody, MABN328, Merck, Watford, UK, 1/1000), anti-PAD2 (ab50257, Abcam, Cambridge, UK, 1/1000), anti-PAD3 (ab50246, 1/1000), all of which have previously been validated in *Gallus gallus* [16] and shown to cross-react with PAD homologues and deiminated proteins from a range of taxa [6,7,9,10,11], as well as the two following EV-specific markers, validated across a wide range of species: CD63 (ab216130, 1/1000; intracellular vesicle marker) and Flotillin-1 (ab41927, 1/2000; specific for the membrane-associated protein caveolae) [8,9,10,11,34,35]. The membranes were thereafter washed in TBS-T for 3 × 10 min at RT and incubated in the corresponding secondary antibody (HRP conjugated anti-rabbit IgG BioRad or anti-mouse IgM, BioRad, diluted 1/4000 in TBS-T) for 1 h, at RT. The membranes were washed for 5 × 10 min in TBS-T and visualisation was performed using enhanced chemiluminescence (ECL) (Amersham, UK) in conjunction with the UVP BioDoc-ITTM System (Thermo Fisher Scientific, Hemel Hempstead, UK).

### 2.5. Immunoprecipitation and Protein Identification

Total deiminated proteins were isolated by immunoprecipitation from plasma of the following three species, representing three taxonomic families: wandering albatross (Diomedeidae), northern giant petrel (Procellariidae) and south polar skua (Stercorariidae). The Catch and Release^®^ v2.0 immunoprecipitation kit (Merck, Watford, UK) was used together with the F95 pan-deimination antibody (MABN328, Merck), which has been developed against a deca-citrullinated peptide and specifically detects proteins modified by citrullination/deimination [74]. For F95 enrichment, 50 μL of plasma was used from each bird and immunoprecipitation was carried out on a rotating platform overnight at 4 °C, according to the manufacturer’s instructions (Merck). The F95 bound proteins were eluted using denaturing elution buffer (Merck), according to the manufacturer’s instructions, and thereafter analysed by Western blotting and by liquid chromatography with tandem mass spectrometry (LC–MS/MS) (Cambridge Proteomics, Cambridge, UK). For LC–MS/MS, the F95-enriched eluates were run 0.5 cm into a 12% TGX gel (BioRad) and each cut out as one band. The 1D gel bands were transferred into a 96-well PCR plate. The bands were cut into 1 mm^2^ pieces, destained, reduced (DTT) and alkylated (iodoacetamide) and subjected to enzymatic digestion with trypsin overnight at 37 °C. After digestion, the supernatant was pipetted into a sample vial and loaded onto an autosampler for automated LC–MS/MS analysis. All LC–MS/MS experiments were performed using a Dionex Ultimate 3000 RSLC nanoUPLC (Thermo Fisher Scientific Inc., Waltham, MA, USA) system and a QExactive Orbitrap mass spectrometer (Thermo Fisher Scientific Inc., Waltham, MA, USA). Separation of peptides was performed by reverse-phase chromatography at a flow rate of 300 nL/min and a Thermo Scientific reverse-phase nano Easy-Spray column (Thermo Scientific PepMap C18, 2 µm particle size, 100 A pore size, 75 µm i.d. × 50 cm length). Peptides were loaded onto a pre-column (Thermo Scientific PepMap 100 C18, 5 µm particle size, 100 A pore size, 300 µm i.d. × 5 mm length) from the Ultimate 3000 autosampler with 0.1% formic acid for 3 min at a flow rate of 10 µL/min. After this period, the column valve was switched to allow elution of peptides from the pre-column onto the analytical column. Solvent A was water + 0.1% formic acid and solvent B was 80% acetonitrile, 20% water + 0.1% formic acid. The linear gradient employed was 2–40% B in 30 min. The LC eluant was sprayed into the mass spectrometer by means of an Easy-Spray source (Thermo Fisher Scientific Inc.). All *m*/*z* values of eluting ions were measured in an Orbitrap mass analyzer, set at a resolution of 70,000 and was scanned between *m*/*z* 380 and 1500. Data dependent scans (Top 20) were employed to automatically isolate and generate fragment ions by higher energy collisional dissociation (HCD, NCE:25%) in the HCD collision cell and measurement of the resulting fragment ions was performed in the Orbitrap analyser, set at a resolution of 17,500. Singly charged ions and ions with unassigned charge states were excluded from being selected for MS/MS and a dynamic exclusion window of 20 s was employed. Post-run, the data was processed using Protein Discoverer (version 2.1., Thermo Scientific). Briefly, all MS/MS data were converted to mgf files and the files were then submitted to the Mascot search algorithm (Matrix Science, London, UK) and due to low annotation of species-specific databases the hit search was carried out against the UniProt Aves database: CCP_Aves_class Aves_class_20190709 (876,224 sequences; 364,491,521 residues) and a common contaminant sequences database (123 sequences; 40,594 residues). The peptide and fragment mass tolerances were set to 20 ppm and 0.1 Da, respectively. A significance threshold value of *p* < 0.05 and a peptide cut-off score of 20 were also applied.

Search Tool for the Retrieval of Interacting Genes/Proteins (STRING) analysis (https://string-db.org/) was used for the identification of putative protein–protein interaction networks for the deiminated proteins identified in northern giant petrel, south polar skua and wandering albatross. Due to lack of species-specific proteins in the STRING database, protein-interaction network analysis was based on human protein identifiers. Protein networks were built by using the function of “search multiple proteins” in STRING and applying basic settings and medium confidence, with colour lines between nodes indicating evidence-based interactions for network edges as follows: known interactions (based on curated databases, experimentally determined), predicted interactions (based on gene neighbourhood, gene fusion, gene co-occurrence) or via text mining, co-expression or protein homology. Coloured nodes in the analysis represent query proteins and first shell of interactors; white nodes represent second shell of interactors.

### 2.6. Statistical Analysis

Histograms and Nanosight graphs were prepared using GraphPad Prism version 7 (GraphPad Software, San Diego, CA, USA) and the Nanosight NS300 software (Malvern, UK). Histograms represent mean of data and standard error of mean (SEM) is indicated by the error bars.

## 3. Results

### 3.1. Extracellular Vesicle Analysis in Seabird Plasma

**Figure 1:**
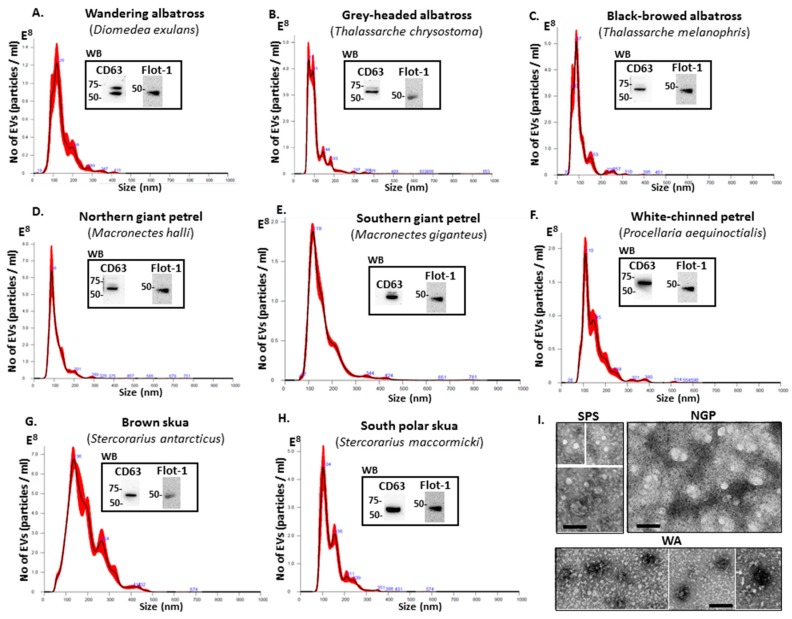
Extracellular vesicle (EV) profiles of seabird plasma. (**A**–**H**) Nanosight particle tracking analysis (NTA) and Western blotting analysis (WB) of EVs isolated from the 8 bird plasma shows some variation in EV size distribution profiles as represented by the histograms and positive immunoblotting with two phylogenetically conserved EV-specific protein markers, CD63 and Flot-1. (**A**) Wandering albatross (*Diomedea exulans*); (**B**) Grey-headed albatross (*Thalassarche chrysostoma*); (**C**) Black-browed albatross (*Thalassarche melanophris*); (**D**) Northern giant petrel (*Macronectes halli*); (**E**) Southern giant petrel (*Macronectes giganteus*). (**F**) White-chinned petrel (*Procellaria aequinoctialis*); (**G**) Brown skua (*Stercorarius antarcticus*); (**H**) South polar skua (*Stercorarius maccormicki*); (**I**) Transmission electron microscopy (TEM) composite images represent examples of EVs isolated from wandering albatross (WA), northern giant petrel (NGP) and south polar skua (SPS); scale bars indicate 100 nm for all images.

EVs from the individual seabird plasma were characterised by size exclusion using NTA (Figure 1A–H), by Western blotting using EV-specific protein markers (Figure 1A–H) and by morphological analysis using transmission electron microscopy (TEM) (Figure 1I). A poly-dispersed population of EVs, overall in the size range of 30 to 500 nm, was observed in plasma of all eight individuals/species, with some differences observed in size distribution profiles (Figure 1A–H). The main EV peaks in plasma were as follows: wandering albatross (48, 120, 198, 280, 347 and 413 nm); grey-headed albatross (72, 93, 144, 183, 297 and 355 nm); black-browed albatross (70, 87, 153, 257 and 310 nm); northern giant petrel (88, 201 and 289 nm); southern giant petrel (67, 119, 344 and 424 nm); white-chinned petrel (110, 145, 238 and 380 nm); brown skua (136, 264 and 432 nm); south polar skua (104, 156, 211 and 239 nm); (Figure 1A–H). Western blotting analysis confirmed that the plasma EVs isolated from all 8 species were positive for the EV-specific markers CD63 and Flot-1 (Figure 1A–H, see inserted WB figures).

**Figure 2:**
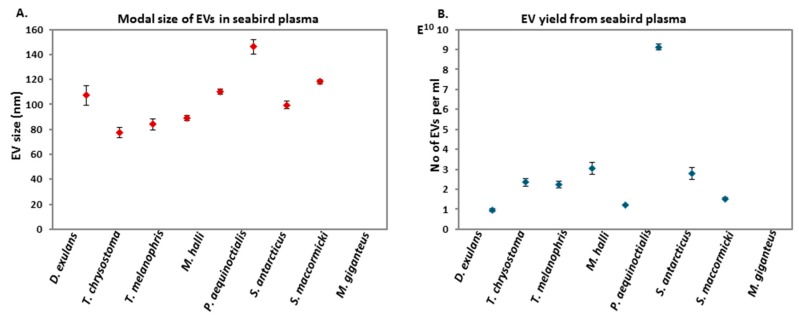
EV modal size and EV yield from plasma of the eight bird species. (**A**) Modal size of plasma-derived EVs varied between bird species but was overall in the range of 80–140 nm, with the largest modal size observed in south polar skua (*S. antarcticus*) and the smallest modal EV size in grey-headed albatross (*T. chrysostoma*). (**B**) Total yield of EVs isolated from plasma varied between the eight bird species, with the highest EV yield from south polar skua (*S. antarcticus*), but lowest EV yield from plasma of wandering albatross (*D. exulans*). For each species, EVs were measured in one individual per species, in five 60 s videos; each scatter dot therefore indicates the average of the five repeated readings per sample, and the error bars indicate +/− standard error for these five readings of EV size distribution profile (**A**) and EV yield per sample (**B**).

Comparing the EV profiles, modal size ranged from 80 to 140 nm; the largest EVs were found in the brown skua (Figure 2A). The yield of EVs isolated from the seabird plasma also varied and was highest in the brown skua (9 × 10^10^ particles/mL), while the proportionally lowest EV yield was found in the wandering albatross and white-chinned petrel (1.5 × 10^10^ particles/mL) (Figure 2B).

### 3.2. Deiminated Proteins and PAD in Seabird Plasma

**Figure 3:**
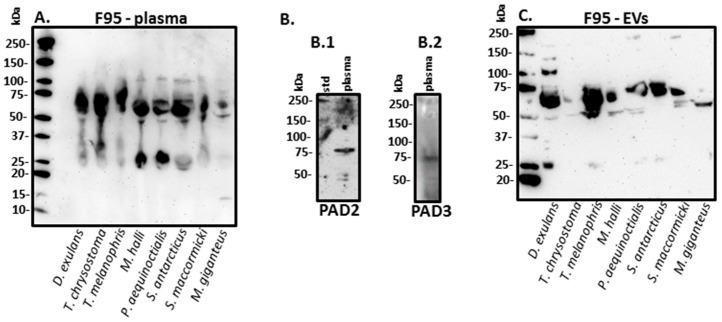
Deiminated proteins in seabird plasma and plasma-derived EVs. (**A**) Deimination positive protein bands, as assessed by the pan-deimination F95 antibody, were observed in plasma of all eight bird species tested in this study, in the size range of 25–150 kDa. (**B**) Peptidylarginine deiminase (PAD) homologues via cross reaction with anti-human PAD2 antibody (**B.1**) and anti-human PAD3 antibody (**B.2**) were observed in seabird plasma at an expected size of approximately 75 kDa. The protein standard (std) is indicated in kilo Daltons (kDa) on the left hand side of each blot. (**C**) Plasma-derived EVs were positive for deiminated proteins, as assessed by the pan-deimination F95 antibody, in plasma-EVs isolated from all eight bird species tested in this study. This confirms EV-mediated export of deiminated proteins.

Total deiminated proteins in the seabird plasma (one representative individual per species) were detected using the F95 pan-deimination antibody, revealing a range of proteins between 10 and 250 kDa by Western blotting analysis (Figure 3A). PAD homologues were identified in seabird plasma by Western blotting (Figure 3B) via cross reaction with anti-human PAD2 and PAD3 antibodies and detected at an expected approximate 75 kDa size (Figure 3(B.1,B.2)). The plasma-derived EVs were positive for deiminated proteins as assessed by Western blotting, using the pan-deimination F95 antibody (Figure 3C), and therefore confirming EV-mediated export of deiminated proteins.

**Figure 4:**
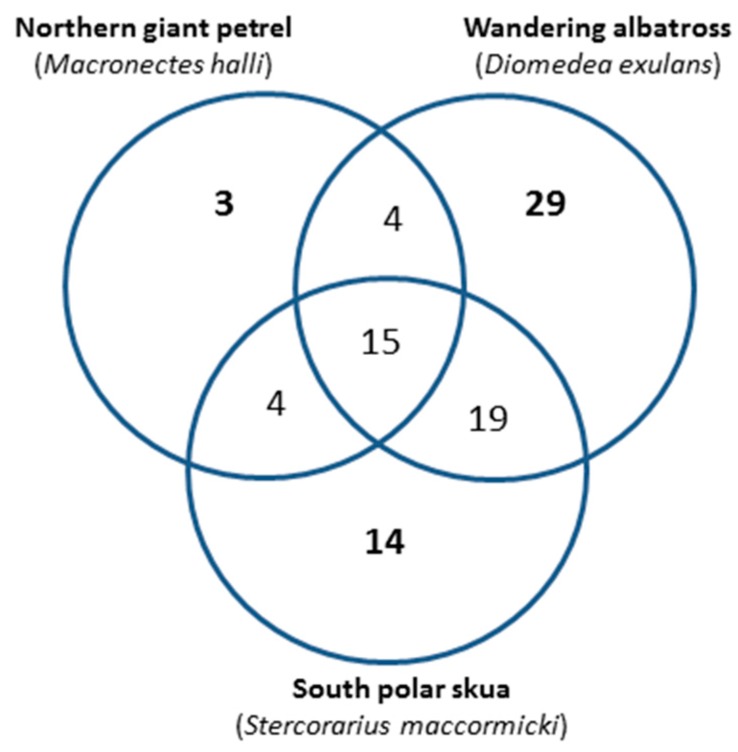
Venn diagram of deiminated protein hits identified in seabird plasma by liquid chromatography with tandem mass spectrometry (LC–MS/MS). The identity of deiminated proteins isolated by F95 enrichment from plasma of wandering albatross (*Diomedea exulans*), northern giant petrel (*Macronectes halli*) and south polar skua (*Stercorarius maccormicki*) was assessed by LC–MS/MS analysis. Some differences in deiminated protein hits were identified, with 3, 29 and 14 unique hits for northern giant petrel, wandering albatross and south polar skua, respectively. Overall, 15 protein hits were identified as common deimination candidates in all three seabird species tested.

Deiminated protein candidates were further identified by liquid chromatography with tandem mass spectrometry (LC–MS/MS) analysis, following F95 enrichment, in three of the bird species under study, with 26, 53 and 67 deimination protein candidate hits (including unidentified protein hits) identified for wandering albatross, northern giant petrel and south polar skua, respectively, whereof 15 hits were shared between all three species (Figure 4).

Details for deiminated protein hits identified in plasma of the three bird species, with homology to the Aves database, are listed in Table 1, Table 2 and Table 3 (and Supplementary Tables S1–S3), respectively.

### 3.3. Protein–protein Network Interaction Analysis for Deiminated Proteins in Seabird Plasma

**Figure 5:**
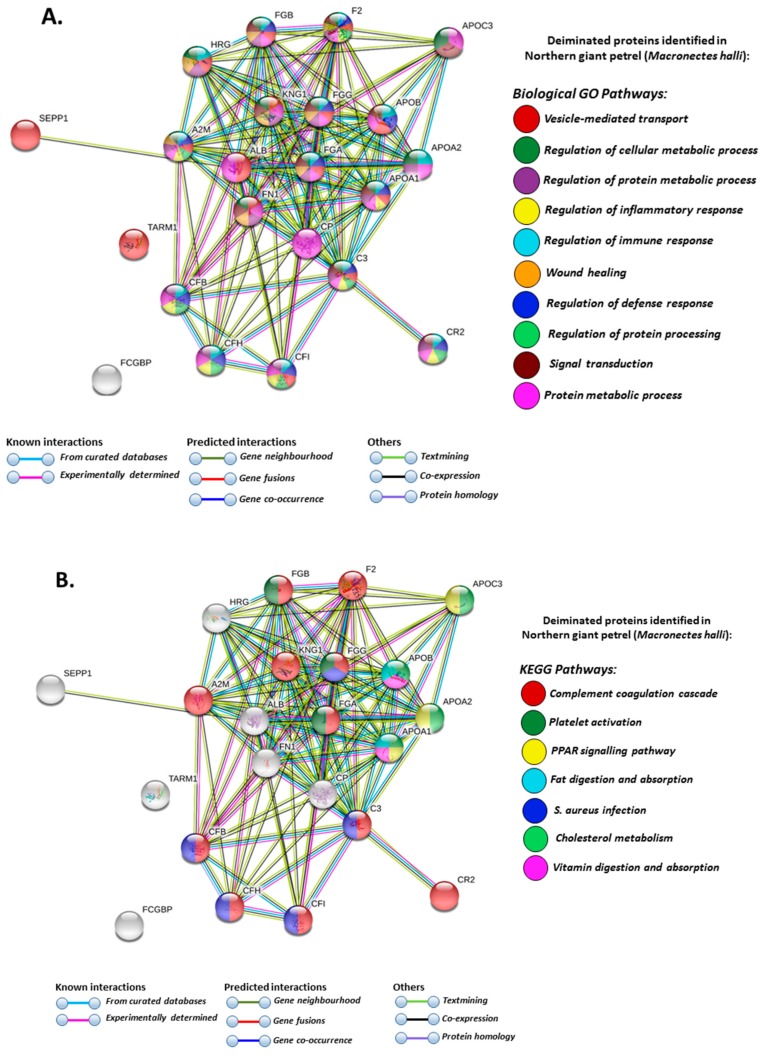
Protein–protein interaction networks of deiminated proteins identified by F95 enrichment in plasma of northern giant petrel (*Macronectes halli*). Reconstruction of protein–protein interactions based on known and predicted interactions of human homologue proteins to proteins identified in northern giant petrel, using Search Tool for the Retrieval of Interacting Genes/Proteins (STRING) analysis. (**A**) Biological Gene Ontology (GO) pathways relating to identified proteins and reported in STRING are highlighted showing vesicle-mediated transport; regulation of cellular metabolic process; regulation of protein metabolic process; regulation of inflammatory response; regulation of immune response; wound healing; regulation of defence response; regulation of protein processing; signal transduction; protein metabolic process. (**B**) Kyoto Encyclopedia of Genes and Genomes (KEGG) pathways relating to the identified proteins and reported in STRING are highlighted showing complement and coagulation cascade; platelet activation; PPAR signalling pathway; fat digestion and absorption; *Staphylococcus aureus* infection; cholesterol metabolism and vitamin digestion and absorption. Coloured nodes represent query proteins and first shell of interactors; white nodes are second shell of interactors. Coloured lines indicate whether protein interactions are identified via known interactions (curated databases, experimentally determined), predicted interactions (gene neighbourhood, gene fusion, gene co-occurrence) or via text mining, co-expression or protein homology (see the colour key for connective lines and for nodes indicating the specific GO and KEGG pathways included in the figure).

**Figure 6:**
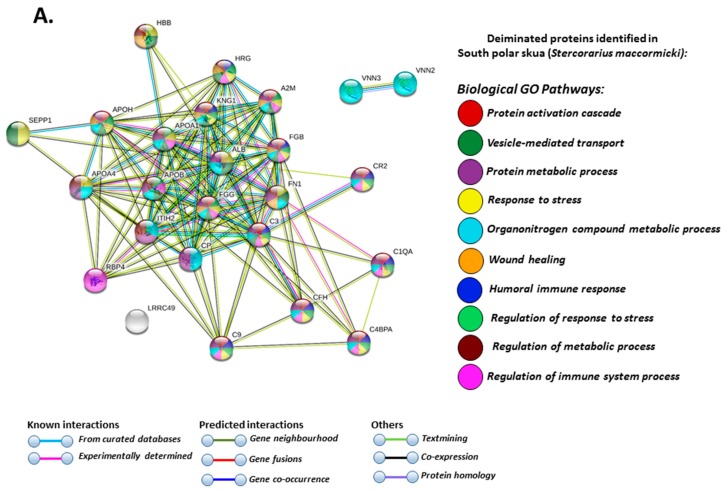

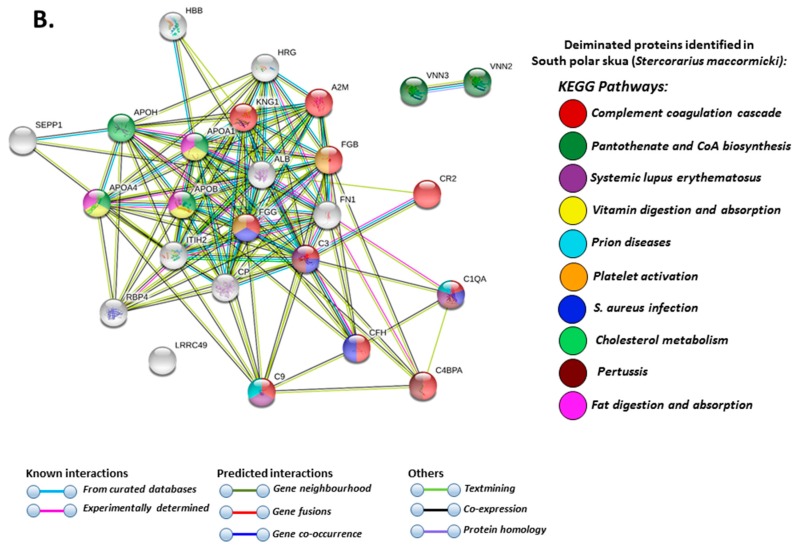
Protein–protein interaction networks of deiminated proteins identified by F95 enrichment in plasma of south polar skua (*Stercorarius maccormicki*). Reconstruction of protein–protein interactions based on known and predicted interactions of human homologue proteins to proteins identified in south polar skua, using STRING analysis. (**A**) Biological GO pathways relating to identified proteins and reported in STRING are highlighted showing protein activation cascade; vesicle-mediated transport; protein metabolic process; response to stress; organonitrogen compound metabolic process; wound healing; humoral immune response; regulation of response to stress; regulation of metabolic process and regulation of immune system process. (**B**) KEGG pathways relating to the identified proteins and reported in STRING are highlighted showing complement and coagulation cascade; pantothenate and CoA biosynthesis; systemic lupus erythematosus; vitamin digestion and absorption; prion diseases; platelet activation; *Staphylococcus aureus* infection; cholesterol metabolism; pertussis and fat digestion and absorption. Coloured nodes represent query proteins and first shell of interactors; white nodes are second shell of interactors. Coloured lines indicate whether protein interactions are identified via known interactions (curated databases, experimentally determined), predicted interactions (gene neighbourhood, gene fusion, gene co-occurrence) or via text mining, co-expression or protein homology (see the colour key for connective lines and for nodes indicating the specific GO and KEGG pathways included in the figure).

**Figure 7:**
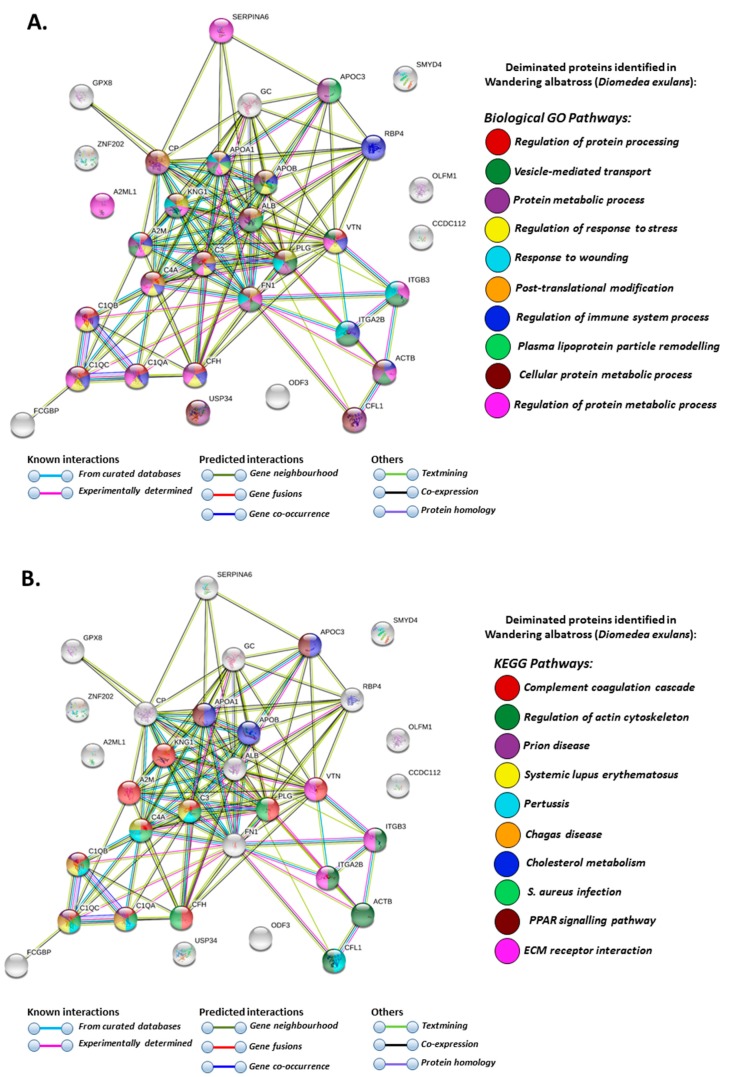
Protein–protein interaction networks of deiminated proteins identified by F95 enrichment in plasma of wandering albatross (*Diomedea exulans*). Reconstruction of protein–protein interactions based on known and predicted interactions of human homologue proteins to proteins identified in wandering albatross, using STRING analysis. (**A**) Biological GO pathways relating to identified proteins and reported in STRING are highlighted showing regulation of protein processing; vesicle-mediated transport; protein metabolic process; regulation of response to stress; response to wounding; post-translational modification; regulation of immune system process; plasma lipoprotein particle remodelling; cellular protein metabolic process and regulation of protein metabolic process. (**B**) KEGG pathways relating to the identified proteins and reported in STRING are highlighted showing complement and coagulation cascade; regulation of actin cytoskeleton; prion disease; systemic lupus erythematosus (SLE); pertussis; Chagas disease; cholesterol metabolism; *Staphylococcus aureus* infection; PPAR signalling pathway; ECM receptor interaction. Coloured nodes represent query proteins and first shell of interactors; white nodes are second shell of interactors. Coloured lines indicate whether protein interactions are identified via known interactions (curated databases, experimentally determined), predicted interactions (gene neighbourhood, gene fusion, gene co-occurrence) or via text mining, co-expression or protein homology (see the colour key for connective lines and for nodes indicating the specific GO and KEGG pathways included in the figure).

Based on Search Tool for the Retrieval of Interacting Genes/Proteins (STRING) analysis, the PPI enrichment *p*-value for the deiminated proteins identified in wandering albatross, northern giant petrel and south polar skua was < 1.0 × 10^−16^ for all three species. Human protein homologues were used for the assessment of the protein interaction networks (Figure 5, Figure 6 and Figure 7) due to a lack of annotations of species-specific bird protein annotations in STRING. For all three seabird species, some of the same biological GO (Gene Ontology) and KEGG (Kyoto Encyclopedia of Genes and Genomes) pathways were identified for deiminated proteins and these included vesicle-mediated transport, protein metabolic processes, response to wounding and wound healing, stress and immune system processes, including complement coagulation cascade, bacterial infection defence pathways (*Staphylococcus aureus*) and cholesterol metabolism (Figure 5, Figure 6 and Figure 7). There were some species-specific differences observed for deiminated protein candidates, as biological GO pathways for signal transduction and KEGG pathways for deiminated proteins involved in fat digestion and absorption were observed in northern giant petrel only (Figure 5A,B).

In south polar skua only, deiminated proteins involved in organonitrogen compound metabolic process were identified, as well as pathways for pantothenate and Coenzyme A (CoA) biosynthesis (Figure 6A,B).

In the wandering albatross only, specific deimination positive pathways were identified involving biological GO pathways for post-translational modification, plasma lipoprotein particle remodelling and KEGG pathways for Chagas disease, regulation of actin cytoskeleton and extracellular matrix (ECM) receptor interaction (Figure 7A,B).

Some pathways were common for two of the species under study, such as KEGG pathways for platelet activation as well as vitamin digestion and absorption for northern giant petrel and south polar skua; while KEGG pathways for prion diseases, systemic lupus erythematousus, pertussis were common to both south polar skua and wandering albatross (Figure 6 and Figure 7); and peroxisome proliferator-activated receptor (PPAR) signalling pathway common for northern giant petrel and wandering albatross (Figure 5 and Figure 7).

## 4. Discussion

The current study describes, for the first time, extracellular vesicle (EV) and deiminated protein profiles in the plasma of a range of Antarctic seabirds, representing three families from two orders (Procellariiformes and Charadriiformes), and two breeding locations (South Georgia and Adelaide Island). Although the analysis was of only one individual from each species (while representing eight species from two avian orders) and therefore the level of intraspecific variation is unknown, our findings nevertheless highlight novel aspects of post-translational deimination in key proteins with functions in innate and adaptive immunity, wound healing and signal transduction, as well as proteins involved in a range of metabolic pathways. As studies on protein deimination in birds are mainly limited to CNS regeneration studies in *Gallus gallus* [16], the current findings provide a first baseline for putative protein moonlighting functions in seabirds via protein deimination. These are hitherto unidentified contributors to different physiological and immunological responses, in the target protein identified in these seabirds, and provide novel insights also into putative species-specific differences. Furthermore, EV profiles in plasma of these seabirds were analysed, showing EVs positive for phylogenetically conserved EV-specific markers and displaying typical EV morphology. We observed some differences in EV size distribution profiles in the diverse seabird species under study, including for example, narrower EV profiles of 50–200 nm in the northern giant petrel, and a higher abundance of larger EVs in the brown skua.

Using antibodies against human PAD2 and PAD3, respectively, peptidylarginine deiminase (PAD) homologues were identified in the seabird plasma by Western blotting for PAD2, which is the phylogenetically most conserved PAD form [1,6], as well as for PAD3, at an expected 70–75 kDa size, similar to that observed for mammalian PADs and *Gallus gallus* PAD3 [16]. This indicates the presence of more than one PAD isozyme in these birds and is in line with previous studies in *Gallus gallus* [1,16]. A range of deiminated proteins identified in the seabird plasma in our study, using F95 enrichment and LC–MS/MS analysis, included key proteins involved in immunity, protein synthesis, response to infection, cell signalling and metabolism.

Based on Search Tool for the Retrieval of Interacting Genes/Proteins (STRING) analysis, the PPI enrichment *p*-value for the deiminated proteins identified in northern giant petrel, south polar skua and wandering albatross, respectively, was <1.0 × 10^−16^ for all three species. Such an enrichment value indicates that the identified network of proteins has significantly more interactions than expected for a random set of proteins of similar size, drawn from the genome. Such an enrichment indicates that the proteins as a group are at least partly connected in terms of their biological function. For all three seabird species assessed, some of the same biological GO (Gene Ontology) and KEGG (Kyoto Encyclopedia of Genes and Genomes) pathways were identified for deiminated proteins; however, some species-specific differences between the three birds for GO and KEGG pathways were also observed (Figure 5, Figure 6 and Figure 7). This indicates that target proteins for deimination may differ between species and possibly contribute to a range of physiological functions including metabolism and immunity, as well as to some key defence mechanisms. Such post-translational deimination may play important roles for their moonlighting roles in physiological and pathophysiological processes. Shared proteins identified to be deiminated in all three species of seabirds assessed were serum albumin, apolipoprotein A-I, fibrinogen, kininogen-1, alpha-2-macroblogulin, complement C3, Factor H, comlement C1q, immunoglobulin, ceruloplasmin, fibronectin, ovotransferrin, alpha-1-antiproteinase 2 and selenoprotein P. Main proteins identified here, and their key roles in immunity and metabolism, are as follows and discussed in relation to Aves where information is available, as well as from a comparative angle with regard to human pathologies. Shared hits between the three seabird species are listed first:

**Serum albumin** is a known glycoprotein in some species and is a major acidic plasma protein in vertebrates and serves as a transport molecule for fatty acids, bilirubin, steroids, amino acids and copper, as well as having roles in maintaining the colloid osmotic pressure of blood [75]. Albumin belongs to acute phase proteins that have been studied in birds, particularly chicken (*Gallus gallus*) [67].

**Apolipoprotein A-I, Apolipoprotein AI-V and Apolipoprotein B-100** were all found to be deiminated in seabird plasma. Apolipoprotein A-I is primarily involved in lipid metabolism and associated with regulation of mitochondrial function and bioenergetics [76,77]. ApoA-I has a regulatory role in the complement system in various species [78,79,80]. ApoA-IV is a lipid binding protein, primarily synthesized in the small intestine and involved in a range of physiological proteins including lipid absorption and metabolism, glucose homeostasis, platelet aggregation and thrombosis [81]. ApoB-100 is synthesised by the liver, plays a part in innate immune responses [82] (Peterson et al., 2008), and is associated with endoplasmic reticulum (ER) stress and insulin resistance [83], as well as lipid metabolism disorders [84].

**Fibrinogen** is a glycoprotein, synthesised in liver and forms part of the acute phase response as part of the coagulation cascade [85]. Fibrinogen has diverse functions, including roles in the immune defence and has for example been associated with host defences against pathogens, as well as in acute phase and stress responses and in toxicity [86,87]. In humans, various fibrinogen disorders are known, related to coagulopathies, ischemic stroke, cancer, liver disease or post-translational modifications [88]. Fibrinogen is a known deimination candidate and this post-translational modification contributes for example to its antigenicity in autoimmune diseases [89,90]. In birds, fibrinogen is a known acute phase protein, and particularly studied in chickens, also in response to immune challenge and infection [67].

**Kininogen-1** forms part of the acute phase response. In mammals, elevated levels of kininogen are linked to sepsis [91]. Roles in inflammatory and oxidative stress pathways have also been described [92].

**Alpha-2-macroglobulin** forms part of the innate immune system and clears active proteases from tissue fluids [93]. Alpha-2-M is phylogenetically-conserved from arthropods to mammals and is closely related to other thioester-containing proteins, complement proteins C3, C4 and C5 [94,95,96].

A range of **complement components** was deiminated in the plasma of our study species, including complement components C3, C4 and C9, which are central to the the alternative and classical pathways and participate in formation of the membrane attack complex (MAC). Furthermore, regulatory factors of the complement system, receptors and recognition molecules, including Factor H, C4b-binding protein, complement receptor type 2, Complement C1q tumour necrosis factor-related protein 3 isoform A and C4b-binding protein alpha chain were also deiminated. These complement proteins are involved both in the alternative and classical complement pathway and contribute to cell lysis as well as being implicated in clearance of apoptotic cells, tissue remodelling and host-defences against pathogens and in infection [7,97,98,99,100]. The identification of post-translational modification of these complement components, which include central complement components, recognition molecules and complement regulatory proteins, is of considerable interest in the light of the multifaceted functions of complement components and the diversification of the complement system throughout phylogeny [98,101,102]. Recently such post-translational deimination of a range of complement components was recognized in both bony and cartilaginous fish [6,7,9,35]. There was a difference in complement component deimination hits observed between the seabird species assessed here, as C1q, C3 and Factor H were identified in all three bird species, while C4, which belongs to the classical pathway, was only found deiminated in wandering albatross, and C9, which forms part of the final complement lysis MAC complex, was only found deiminated in south polar skua. Complement receptor type 2 was found deiminated in northern giant petrel and south polar skua, but not in wandering albatross, while C4b-binding protein was found deiminated in south polar skua only. The role of post-translational deimination for complement protein moonlighting in birds, and its contribution to diverse functions of the complement system in physiological and pathophysiological processes, as well as complement diversification throughout phylogeny remains to be elucidated.

A range of **immunoglobulins** was found to be deiminated in all three seabird species tested. This included IgGFc-binding protein, Ig lambda-1 chain C regions, Ig gamma-1 chain C region, membrane-bound form and Ig heavy chain V-III region KOL. Ig’s are key molecules in adaptive immunity and studied in diverse taxa. Post-translational deimination of Ig’s and roles in Ig function have hitherto received little attention except in teleosts and cartilaginous fish [6,7,9], as well as in the llama *Lama glama* [10] and cetaceans [24]. In human patients with bronchiectasis and RA, the IgG Fc region is post-translationally deiminated [103]. Given the increased interest in furthering understanding of Ig diversity throughout the phylogenetic tree [104,105,106,107] our current finding of deimination of bird Ig’s highlights a novel concept of diversification of Ig function via post-translational deimination.

**Ceruloplasmin** was found to be deiminated in all three seabird species tested. It is a serum ferroxidase with antioxidative function and highly conserved throughout vertebrate evolution. It carries the majority of copper in plasma and has roles in iron homeostasis [108,109]. In birds it has been identified as an inflammatory marker associated with trauma and infection [110] and studied as an acute-phase protein biomarker in broiler breeding lines [67,70].

**Fibronectin** is an important part of the extracellular matrix and is a hepatic glycoprotein protein which constitutes a major protein component of blood plasma. It has major roles in cell migration, differentiation, migration and growth and plays important roles in wound healing, as well as in embryogenesis [111,112]. Fibronectin is associated with a number of pathologies, including cancer and fibrosis [113]. Fibronectin has been previously found to be deiminated in various sites, which has been related to autoimmunity [114], and also found to support wound healing [115]. In birds, fibronectin is an acute phase protein in chickens (*Gallus gallus*), responding to infection and changes in temperature [67].

**Ovotransferrin** is an iron-binding glycoprotein, belonging to the transferring family of iron-binding glycoproteins. In birds, ovotransferrin is the only form and present in both plasma and egg albumen, while in mammals two forms of transferrin (serum transferrin and lactoferrin), with different functions exist [116]. As ovotransferrin has multifaceted functions and plays major roles in avian natural immunity [67,70,116,117], post-translational deimination may contribute to its diverse functions.

**Alpha-1-antiproteinase 2** belongs to the serpin superfamily, is a protease inhibitor protecting tissues from enzymes of inflammatory cells, and an acute-phase protein, levels of which rise upon acute inflammation [118,119,120]. While it is a known glycoprotein [121], post-translational deimination has not been reported before.

**Selenoprotein P** (Sepp1) is a plasma glycoprotein, mainly secreted from liver but also other tissues and contains most of the selenium in plasma [122]. It has antioxidant properties [122] and serves in homeostasis and distribution of selenium [123]. In birds, selenoprotein has been shown to be important in immune responses [124] and to be protective against growth inhibition, including nutritional muscular dystrophy [125], as well as oxidative damage and apoptosis in response to fluorine [126]. Phylogenetically, Sepp1 is believed to have appeared in early metazoan species [127]. While Sepp1 is known to be glycosylated, little is understood about roles for post-translational deimination for its function.

**Hemoglobin** was found deiminated in northern giant petrel and south polar skua plasma. It is a key molecule in molecular oxygen transport in the bloodstream. In the south polar skua, two haemoglobins had peculiar functional features including additional phosphate binding sites, possible as an adaption to extreme environmental conditions [128,129]. Post-translational modifications, including deimination identified here, may further add to such functional adaptions.

**Vitamin D-binding protein** was found deiminated in wandering albatross plasma. It is a multifaceted protein mainly produced in the liver, where its regulation is influenced by oestrogen, glucocorticoids and inflammatory cytokines [130]. It is secreted into the blood circulation and is able to bind the various forms of vitamin D [131]. It is at higher levels in geese during the laying than pre-laying period, indicative of roles in lipid metabolism related to egg formation [132]. In humans, VDBP is implicated in cancer and coronary artery disease [133,134]. VDBP has previously been identified to be glycosylated [135] and post-translational deimination identified here may further add to its functional diversity.

**Vitronectin (VTN)** was found deiminated in wandering albatross plasma. It is a glycoprotein of the hemopexin family, which is abundant in serum, the extracellular matrix and in bone. In mammals, VTN is a key controller of tissue repair and remodelling activity [136]. It promotes cell adhesion and spreading, and furthermore inhibits the membrane-damaging effect of the terminal cytolytic complement pathway and binds to several serine protease inhibitors [137,138]. Roles for VTN in haemostasis and tumour malignancy have also been described [139,140].

**Noelin** (olfactomedin 1 or pancortin) was found deiminated in wandering albatross plasma. It is a member of the olfactomedin domain-containing superfamily and a highly expressed neuronal glycoprotein important for nervous system development [141]. It binds a range of secreted proteins and cell surface-bound receptors for induction of cell signalling processes and its structure has been described in detail [142]. Noelin also plays important roles in synaptic plasticity [143]. It is also related to growth and metastasis suppression of colorectal cancer [144] and linked to epithelial-mesenchymal transition in the chick embryonic heart [145]. Deimination of noelin identified here has not been studied before and provides a new aspect of multifaceted functions of noelin via such post-translational modification.

**Histidine-rich glycoprotein** was found deiminated in plasma of northern giant petrel and south polar skua. It is a multifaceted glycoprotein which is synthesised in the liver and also white blood cells [146] and is located in plasma and platelets, where it binds amongst other heme and metal ions [147]. It has numerous biological functions including in immunity, vascularisation and coagulation [148,149]. Due to roles in angiogenesis, which can be both pro- and anti-angiogenic, it is also implicated in cancer [150]. Furthermore, it also plays roles in infection and has selective antibacterial activity [151].

**Protein NEL** also known as protein kinase C-binding protein, was found deiminated in south polar skua plasma only. It has a broad array of cellular functions [152]. It is a cytoplasmic glycoprotein involved in cell growth regulation and differentiation, and roles in neural function and development have been described in the chick [153,154].

**Plasma serine protease inhibitor** was found deiminated in south polar skua plasma. It belongs to the serpins, which have multifaceted roles via protease inhibition activity, including in blood clotting, inflammatory and immune responses [119,155,156]. As the protease inhibitor effects of serpins are achieved through conformational changes, also involving beta-sheets [157], this may be of considerable interest as beta-sheets belong to structures prone to post-translational deimination [2].

**Glutathione peroxidase** was found deiminated in south polar skua and wandering albatross plasma. It forms part of the glutathione (GSH) biosynthesis pathway involved in homeostasis and cellular maintenance and also acts as a potent antioxidant [158].

**Pantetheinase**, also known as non-inflammatory molecule-1 (vanin 1), was found deiminated in south polar skua plasma. It has physiological roles related to coenzyme A (CoA) metabolism, lipid metabolism, and energy production [159,160]. It also has a range of roles in relation to oxidative stress and inflammation in developmental, repair and inflammatory processes, contributing to tissue tolerance to stress, and is related to a range of human pathologies [160,161].

**Vascular non-inflammatory molecule 3** also belongs to the vanin family of encoding pantetheinase isoforms and was found deiminated in south polar skua plasma. It has physiological roles in metabolising proteins, carbohydrates and fats [162]. It also has roles in inflammatory pathways via neutrophils and the induction of proinflammatory cytokines [163,164], and has been identified as a biomarker in acute graft-versus-host disease [165].

**Beta-2-glycoprotein 1 (β2GPI)** was found deiminated in south polar skua plasma. It is a circulating blood protein with several essential physiological roles, including in haemostasis, homeostasis and immunity [166]. It furthermore is associated with autoimmunity [167] and has anti-bacterial effects [168,169]. The diverse function of β2GPI, including its dual capability to up- and down-regulate the complement and coagulation systems depending upon external stimulus [170] may reflect hitherto unrecognised structural modifications via post-translational deimination, and be related to glycolysis [167].

**Inter-alpha-trypsin inhibitor** was found deiminated in south polar skua plasma. It is an acute inflammatory marker [171] which functions as a protease inhibitor and is linked to a range of inflammatory responses [172], oxidative stress [173] and infection [174]. Furthermore, inter-α inhibitor proteins play roles in maintaining the resting state of neutrophils by regulating shape and reducing ROS production [175].

**Leucine-rich repeat-containing protein 49** was here identified as post-translationally deiminated in south polar skua plasma. Leucine-rich repeat-containing proteins are linked to a range of functions including mitochondrial transcription [176] and inflammatory responses [177].

**Deleted in malignant brain tumours 1 protein** was found deiminated in wandering albatross plasma. It is a glycoprotein containing multiple scavenger receptor cysteine-rich (SRCR) domains and is related to cellular immune defences and mucosal immunity, as well as to regeneration [178,179,180]. It has dual functions in viral transmission [181] and displays a broad calcium-dependent binding spectrum against a range of bacterial pathogens [180,182]. Absence of the protein or glycosylation has been described in cancer [183,184,185,186], while roles for post-translational deimination in its multifaceted functions remain unknown. This may be of particular interest as DMBT1 shows a pattern recognition activity for poly-sulfated and poly-phosphorylated ligands, including nucleic acids, and also has the ability to aggregate ligands—properties which have made it a protein of interest for targeted nano-delivery [187]. Therefore, indication of structural changes of this protein via post-translational deimination may be of considerable interest.

**Soluble scavenger receptor cysteine-rich domain-containing protein ssc5d-like** was found deiminated in wandering albatross plasma. It plays a role in the innate defence and homeostasis [188]. It binds to extracellular matrix proteins and acts as a pattern recognition receptor (PRR) by binding to pathogen-associated molecular patterns (PAMPs) present on the cell walls of bacteria and fungi, subsequently inhibiting PAMP-induced cytokine release [189]. It is implicated in arthritis [190].

**Corticosteroid binding globulin (CBG)** was found deiminated in wandering albatross plasma. It is the primary cortisol binding protein capable of conformational change from a high cortisol-binding affinity form to a low affinity form [191]. The main role of CBG is in acute, severe inflammation where depletion is associated with mortality, and to chronic inflammation where defects in cortisol delivery may perpetuate inflammation [191,192]. Furthermore, it has roles in metabolism and neurocognitive function, implying that CBG is a multifaceted component in the mechanisms of hypothalamic-pituitary-adrenal axis related homeostasis [191]. In free-living birds, corticosterone may be involved in delaying the onset of breeding including via altering hormone titers, negative feedback regulation, plasma binding globulin concentrations, intracellular receptor concentrations, enzyme activity and interacting hormone systems [193]. It is also implicated in corticosterone regulation in the songbird brain [194]. Such diverse functions may indeed be facilitated via post-translational changes, including deimination recognised here.

**Retinol-binding protein 4 (RBP4)** was found deiminated in south polar skua and wandering albatross plasma. It is mainly synthesized in the liver and circulates in the bloodstream bound to retinol in a complex with transthyretin. It delivers retinol from the liver stores to the peripheral tissues [195]. RBP4 has recently been described as an adipokine that contributes to insulin resistance and diabetes [196], partly via activation of antigen-presenting cells [197]. RBP4 is also secreted by adipocytes of the fat tissue in a smaller portion and acts as a signal to surrounding cells, when there is a decrease in plasma glucose concentration [198]. While some post-translationally processed forms of human RBP4 have been implicated in in chronic renal failure [199], deimination has not been assessed.

**Ubiquitin carboxyl-terminal hydrolase (UCH)** was found deiminated in wandering albatross plasma. It is a deubiquitinating enzyme, essential for a variety of biological processes including cell growth, differentiation, transcriptional regulation, and oncogenesis [200]. It is highly specific to neurones and to cells of the diffuse neuroendocrine system, required for the maintenance of axonal integrity, and its dysfunction is implicated in neurodegenerative disease [201]. Furthermore, it is a reliable serum biomarker for outcome prediction in traumatic brain injury [202]. UCH also plays roles in protecting neurones against ZnO particle-induced neurotoxicity via modulation of the NF-κB signalling pathway [203]. While UCH has been found to have low expression in other healthy tissues, it is highly expressed in several forms of cancer. Interestingly, UCH enzymes can act both as a tumour suppressor and tumour promotor and influence several signalling pathways that play crucial roles in oncogenesis, tumour invasion, and migration [200]. There are also indications that UCHL1 contributes to metabolic response following thermal injury [204]. In birds, UCH enzymes have been described in chick muscle [205]. To our knowledge, post-translational deimination of UCH enzymes has not been described and may well contribute to the moonlighting functions of these hydrolases.

**Collagen alpha-4 (VI) chain** was found deiminated in wandering albatross plasma. It is an extracellular matrix protein [206], is found in lymphoid tissues [207] and has roles, amongst others, in cellular and mucosal immunity and inflammatory diseases such as ulcerative colitis and membranous glomerulonephritis [208,209]. In birds, it has roles in CNS development relating to plasticity and axon growth in the chicken [210].

**SET and MYND domain-containing protein 4** was found deiminated in wandering albatross plasma. It belongs to the Smyd Family of Methyltransferases, which is recognized in diverse taxa [211]. SMYD can methylate histones and non-histone proteins and have diverse roles in chromatin remodelling as well as normal development, in cell growth and differentiation and in the regulation of a series of pathophysiological processes, including cardiac and skeletal muscle physiology and pathology and cancer [212,213,214]. As PADs cause deimination of several histones, the deimination of histone regulatory proteins, such as SMYD here, may be of considerable interest, particularly in the light of their multifaceted functions in regulating a range of histone and non-histone proteins, including histone H3 in *Gallus gallus* [16].

The current study describes for the first time post-translational deimination of a range of proteins involved in immunological and metabolic pathways in pelagic seabirds in the Antarctic. Besides novel insights into diverse protein functions through post-translational modifications in bird physiology, our findings also further knowledge of the translatable functions of PADs throughout the phylogenetic tree, informing comparative studies. Given the numerous and complex structural and functional changes that proteins can undergo via various post-translational modifications, the roles for post-translational deimination in protein moonlighting during physiological and pathophysiological processes, are a promising field for further studies. Similarly, roles for EV-mediated cellular communication in different animal groups is currently an expanding field of research.

## 5. Conclusions

Our findings unravel hitherto unrecognised biomarkers in Antarctic seabirds, which are likely to be indicative of immunological and metabolic functions, and possibly health status. The EV and deimination profiles generated in this study provide a suite of novel biomarkers with considerable potential for developing novel tools to assess seabird health status, as well as providing insights into phylogenetically conserved mechanisms in cellular communication via EV-mediated transport, further informing EV-mediated pathologies. Future research would involve assessing EV cargo, including deiminated EV cargo, in addition to overall plasma deimination biomarkers, in Antarctic seabirds at the individual level, in relation to environmental conditions, pollutant levels and past or recent immune challenges from pathogens. In addition, wider sampling within and between populations would provide further insights into effects of environmental variation, enabling comparisons with normal physiological protein deimination status and EV profiles. This would be particularly valuable for assessing natural and anthropogenic stresses in seabirds in general, many of which are declining and face increasing threats both on land and at sea related to changing climate [41]. While the current study lays a baseline for these novel biomarkers, future studies will need to further refine and develop these markers as an applicable tool in the evaluation of seabirds’ health status.

## Figures and Tables

**Table 1:** Deiminated protein hits identified by F95 enrichment in plasma of northern giant petrel (*Macronectes halli*). Deiminated proteins were isolated by immunoprecipitation using the pan-deimination F95 antibody. The F95-enriched eluate was analysed by LC–MS/MS and peak list files were submitted to mascot. Peptides matching with Aves_class_20190709 (876,224 sequences; 364,491,521 residues) are shown and total score is reported. Protein hits with Aves are indicated, including species name. Protein hits which were identified as deiminated in northern giant petrel only, and not in wandering albatross or south polar skua are listed first and highlighted in light green and with an asterix (*). For full LC–MS/MS data analysis, see Supplementary Table S1.

Protein Name	Species Name	Common Name	Total Score (*p* < 0.05) ^ⱡ^
* A0A093J7B4_FULGA *Myeloid protein 1*	*Fulmarus glacialis*	Northern fulmar	196
* A0A091UTV5_NIPNI *Ig lambda-1 chain C regions*	*Nipponia nippon*	Japanese crested ibis	155
* A0A2P4TBI3_BAMTH *Uncharacterized protein*	*Bambusicola thoracicus*	Chinese bamboo partridge	121
A0A093IER0_FULGA *Fibrinogen beta chain*	*Fulmarus glacialis*	Northern fulmar	1102
A0A093INM3_FULGA *Fibrinogen alpha chain*	*Fulmarus glacialis*	Northern fulmar	1060
A0A1V4JT39_PATFA *Fibrinogen beta chain*	*Patagioenas fasciata monilis*	Band-tailed pigeon (western)	924
A0A0Q3PZX3_AMAAE *Fibrinogen gamma chain*	*Amazona aestiva*	Turquoise-fronted parrot	916
A0A093FHI9_GAVST *Serum albumin*	*Gavia stellata*	Red-throated loon	841
A0A093P0F9_PYGAD *Serum albumin*	*Pygoscelis adeliae*	Adélie penguin	841
A0A0A0A3R1_CHAVO *Apolipoprotein A-I*	*Charadrius vociferus*	Killdeer	786
A0A093LU79_FULGA *Fibronectin*	*Fulmarus glacialis*	Northern fulmar	676
A0A093GBQ7_DRYPU *Fibronectin*	*Dryobates pubescens*	Downy woodpecker	664
A0A0Q3LVM5_AMAAE *Apolipoprotein A-I*	*Amazona aestiva*	Turquoise-fronted parrot	597
A0A087VRD9_BALRE *Serum albumin*	*Balearica regulorum gibbericeps*	Grey crowned crane	596
A0A091SMJ2_PELCR *Serum albumin*	*Pelecanus crispus*	Dalmatian pelican	585
A0A087R4G9_APTFO *Alpha-2-macroglobulin*	*Aptenodytes forsteri*	Emperor penguin	572
A0A093KX01_FULGA *Alpha-2-macroglobulin*	*Fulmarus glacialis*	Northern fulmar	550
A0A091KH67_9GRUI *Serum albumin*	*Chlamydotis macqueenii*	MacQueen’s bustard	542
A0A093IHU9_FULGA *Fibrinogen gamma chain*	*Fulmarus glacialis*	Northern fulmar	470
A0A091WH83_NIPNI *Serum albumin*	*Nipponia nippon*	Japanese crested ibis	458
A0A1V4JT04_PATFA *Fibrinogen gamma chain*	*Patagioenas fasciata monilis*	Band-tailed pigeon (western)	437
A0A091PM78_LEPDC *Apolipoprotein A-I*	*Leptosomus discolor*	Cuckoo roller	432
A0A093KM83_FULGA *Ovotransferrin*	*Fulmarus glacialis*	Northern fulmar	431
A0A2I0UMY8_LIMLA *Fibrinogen gamma chain*	*Limosa lapponica baueri*	Bar-tailed godwit	428
A0A087RJ23_APTFO *Kininogen-1*	*Aptenodytes forsteri*	Emperor penguin	398
A0A091PXP6_HALAL *Fibrinogen alpha chain*	*Haliaeetus albicilla*	White-tailed eagle	368
A0A093CUQ3_9AVES *Fibrinogen alpha chain*	*Pterocles gutturalis*	Yellow-throated sandgrouse	367
A0A091I8G9_CALAN *Serum albumin*	*Calypte anna*	Anna’s hummingbird	353
A0A093PBF1_PYGAD *Alpha-2-macroglobulin*	*Pygoscelis adeliae*	Adélie penguin	346
U3K0Q3_FICAL *Serum albumin*	*Ficedula albicollis*	Collared flycatcher	344
R7VRC4_COLLI *Complement C3*	*Columba livia*	Rock dove	337
A0A0Q3US23_AMAAE *Kininogen-1*	*Amazona aestiva*	Turquoise-fronted parrot	330
A0A093NZR4_PYGAD *Kininogen-1*	*Pygoscelis adeliae*	Adélie penguin	325
A0A099ZYE0_CHAVO *Alpha-2-macroglobulin*	*Charadrius vociferus*	Killdeer	318
A0A093ISV2_FULGA *IgGFc-binding protein*	*Fulmarus glacialis*	Northern fulmar	298
A0A093JJA1_STRCA *Apolipoprotein A-I*	*Struthio camelus australis*	South African ostrich	267
G1MPR2_MELGA *Complement C3*	*Meleagris gallopavo*	Wild turkey	264
A0A087RBR7_APTFO *Ceruloplasmin*	*Aptenodytes forsteri*	Emperor penguin	264
A0A099ZCF9_TINGU *Alpha-2-macroglobulin*	*Tinamus guttatus*	White-throated tinamou	259
A0A093FI89_GAVST *Alpha-1-antiproteinase 2*	*Gavia stellata*	Red-throated loon	251
A0A091FFS0_9AVES *Apolipoprotein A-I*	*Cuculus canorus*	Common cuckoo	246
A0A094K5H2_ANTCR *Ceruloplasmin*	*Antrostomus carolinensis*	Chuck-will’s-widow	239
A0A093IJM0_FULGA *IgGFc-binding protein*	*Fulmarus glacialis*	Arctic fulmar	225
A0A493T9F7_ANAPP *Complement C3*	*Anas platyrhynchos platyrhynchos*	Mallard	223
A0A218ULE2_9PASE *Alpha-2-macroglobulin*	*Lonchura striata domestica*	Bengalese finch	191
A0A093TAA7_PHACA *Serum albumin*	*Phalacrocorax carbo*	Great cormorant	185
A0A2I0TTX4_LIMLA *Kininogen-1*	*Limosa lapponica baueri*	Bar-tailed godwit	182
A0A093NV14_PYGAD *Complement factor H*	*Pygoscelis adeliae*	Adélie penguin	155
A0A091HFG6_BUCRH *Complement factor H*	*Buceros rhinoceros silvestris*	Rhinoceros hornbill	154
A0A087R546_APTFO *Alpha-1-antiproteinase 2*	*Aptenodytes forsteri*	Emperor penguin	129
A0A0Q3NFW7_AMAAE *Alpha-1-antiproteinase 2-like protein*	*Amazona aestiva*	Turquoise-fronted parrot	124
A0A091V0T3_NIPNI *IgGFc-binding protein*	*Nipponia nippon*	Japanese crested ibis	122
A0A087QPM6_APTFO *Complement receptor type 2*	*Aptenodytes forsteri*	Emperor penguin	117
A0A0Q3PU08_AMAAE *Ig gamma-1 chain C region, membrane-bound form*	*Amazona aestiva*	Turquoise-fronted parrot	84
A0A087QSZ7_APTFO *Selenoprotein P*	*Aptenodytes forsteri*	Emperor penguin	83
A0A091RRK2_NESNO *Complement C3*	*Nestor notabilis*	Kea	77
R0L2Q3_ANAPL *IgGFc-binding protein*	*Anas platyrhynchos*	Mallard	76
A0A1V4KDF4_PATFA *Complement C1q tumor necrosis factor-related protein 3 isoform A*	*Patagioenas fasciata monilis*	Band-tailed pigeon (western)	75
A0A087V351_BALRE *Ig heavy chain V-III region KOL*	*Balearica regulorum gibbericeps*	Grey crowned crane	73
A0A087R4G1_APTFO *Apolipoprotein B-100*	*Aptenodytes forsteri*	Emperor penguin	69
A0A091J8Z6_EGRGA *Ig heavy chain V-III region VH26*	*Egretta garzetta*	Little egret	61
A0A093DRD7_9AVES *Hemoglobin subunit alpha-A*	*Pterocles gutturalis*	Yellow-throated sandgrouse	55
A0A226NM49_CALSU *Uncharacterized protein*	*Callipepla squamata*	Scaled quail	52
A0A091FXD5_9AVES *Histidine-rich glycoprotein*	*Cuculus canorus*	Common cuckoo	50
A0A2I0TNP2_LIMLA *Selenoprotein pb-like*	*Limosa lapponica baueri*	Bar-tailed godwit	50

^ⱡ^ Ions score is −10. * Log (P), where P is the probability that the observed match is a random event. Individual ions scores > 40 indicated identity or extensive homology (*p* < 0.05). Protein scores were derived from ions scores as a non-probabilistic basis for ranking protein hits.

**Table 2:** Deiminated proteins identified by F95 enrichment in plasma of south polar skua (*Stercorarius maccormicki*). Deiminated proteins were isolated by immunoprecipitation using the pan-deimination F95 antibody. The F95-enriched eluate was analysed by LC–MS/MS and peak list files were submitted to mascot. Peptides matching with Aves_class_20190709 (876,224 sequences; 364,491,521 residues) are listed and total score is reported. Protein hits with Aves are indicated, including species name. Protein hits which were identified as deiminated in south polar skua only, and not in northern giant petrel or wandering albatross are listed first and highlighted in light blue and with an asterix (*). For full LC–MS/MS data analysis, see Supplementary Table S2.

Protein Name	Species Name	Common Name	Total Score (*p* < 0.05) ^ⱡ^
* U3JY34_FICAL *Uncharacterized protein*	*Ficedula albicollis*	Collared flycatcher	403
* A0A091EPY9_CORBR *Protein NEL*	*Corvus brachyrhynchos*	American crow	266
* U3K9W1_FICAL *Uncharacterized protein*	*Ficedula albicollis*	Collared flycatcher	189
* A0A2I0UHP4_LIMLA *Uncharacterized protein*	*Limosa lapponica baueri*	Bar-tailed godwit	182
* A0A2I0U6I0_LIMLA *Complement component c9*	*Limosa lapponica baueri*	Bar-tailed godwit	181
* A0A0A0A0R4_CHAVO *Complement component C9*	*Charadrius vociferus*	Killdeer	159
* A0A091U8P6_PHORB *Complement component C9*	*Phoenicopterus ruber ruber*	American flamingo	157
* A0A087VFS5_BALRE *Plasma serine protease inhibitor*	*Balearica regulorum gibbericeps*	Grey crowned crane	144
* A0A2I0TEM1_LIMLA *C4b-binding protein alpha chain*	*Limosa lapponica baueri*	Bar-tailed godwit	111
* A0A1V4KDF8_PATFA *Complement component C9*	*Patagioenas fasciata monilis*	Band-tailed pigeon (western)	107
* U3JJN2_FICAL *Uncharacterized protein*	*Ficedula albicollis*	Collared flycatcher	99
* U3JJN2_FICAL *Uncharacterized protein*	*Ficedula albicollis*	Collared flycatcher	99
* A0A091EHN6_CORBR *Plasma serine protease inhibitor*	*Corvus brachyrhynchos*	American crow	97
* A0A087R6D3_APTFO *Pantetheinase*	*Aptenodytes forsteri*	Emperor penguin	88
* A0A091NIR3_9PASS *Uncharacterized protein*	*Acanthisitta chloris*	Rifleman	79
* A0A091U4S2_PHORB *Vascular non-inflammatory molecule 3*	*Phoenicopterus ruber ruber*	American flamingo	78
* U3JSQ8_FICAL *Apolipoprotein A4*	*Ficedula albicollis*	Collared flycatcher	74
* A0A087RA43_APTFO *Beta-2-glycoprotein 1*	*Aptenodytes forsteri*	Emperor penguin	69
* A0A087QMI5_APTFO *Inter-alpha-trypsin inhibitor heavy chain H2*	*Aptenodytes forsteri*	Emperor penguin	69
* A0A091KRT6_COLST *Alpha-1-antitrypsin-like GS55-MS*	*Colius striatus*	Speckled mousebird	67
* A0A091KJ46_9GRUI *Ovoinhibitor*	*Chlamydotis macqueenii*	MacQueen’s bustard	66
* A0A087VN55_BALRE *Pantetheinase*	*Balearica regulorum gibbericeps*	Grey crowned crane	56
* A0A091JHT9_EGRGA *Leucine-rich repeat-containing protein 49*	*Egretta garzetta*	Little egret	51
* A0A226PWY7_COLVI *Uncharacterized protein*	*Colinus virginianus*	Northern bobwhite	50
* A0A093BZB9_9AVES *Zinc finger protein 518A*	*Pterocles gutturalis*	Yellow-throated sandgrouse	45
A0A0A0AN62_CHAVO *Serum albumin*	*Charadrius vociferus*	Killdeer	1363
A0A091LDB0_CATAU *Alpha-2-macroglobulin*	*Cathartes aura*	Turkey vulture	1265
A0A0A0A1J2_CHAVO *Alpha-2-macroglobulin*	*Charadrius vociferus*	Killdeer	1259
A0A093F817_TYTAL *Serum albumin*	*Tyto alba*	Barn owl	1225
A0A093RKW8_PYGAD *Alpha-2-macroglobulin*	*Pygoscelis adeliae*	Adélie penguin	1129
A0A1V4JAY4_PATFA *Alpha-2-macroglobulin*	*Patagioenas fasciata monilis*	Band-tailed pigeon (western)	1063
A0A094L652_ANTCR *Serum albumin*	*Antrostomus carolinensis*	Chuck-will’s-widow	961
A0A226MDX7_CALSU *Serum albumin*	*Callipepla squamata*	Scaled quail	930
A0A087VRD9_BALRE *Serum albumin*	*Balearica regulorum gibbericeps*	Grey crowned crane	915
A0A0A0A3R1_CHAVO *Apolipoprotein A-I*	*Charadrius vociferus*	Killdeer	912
A0A091MMC9_CARIC *Serum albumin*	*Cariama cristata*	Red-legged seriema	892
A0A091RWK1_9GRUI *Serum albumin*	*Chlamydotis macqueenii*	MacQueen’s bustard	877
A0A093H6Z2_DRYPU *Apolipoprotein A-I*	*Dryobates pubescens*	Downy woodpecker	863
A0A0Q3X9Z0_AMAAE *Serum albumin-like protein*	*Amazona aestiva*	Turquoise-fronted parrot	840
A0A091TRL5_PHALP *Alpha-2-macroglobulin*	*Phaethon lepturus*	White-tailed tropicbird	820
R0M0W6_ANAPL *Serum albumin*	*Anas platyrhynchos*	Mallard	802
A0A2I0MH12_COLLI *Albumin*	*Columba livia*	Rock dove	758
A0A091MK58_CARIC *Alpha-1-antiproteinase 2*	*Cariama cristata*	Red-legged seriema	754
A0A0Q3LVM5_AMAAE *Apolipoprotein A-I*	*Amazona aestiva*	Turquoise-fronted parrot	749
A0A091G8Y4_9AVES *Serum albumin*	*Cuculus canorus*	Common cuckoo	718
A0A2I0UH92_LIMLA *Alpha-1-antiproteinase 2-like*	*Limosa lapponica baueri*	Bar-tailed godwit	716
A0A091PEU7_LEPDC *Fibronectin*	*Leptosomus discolor*	Cuckoo roller	695
A0A091KH67_9GRUI *Serum albumin*	*Chlamydotis macqueenii*	MacQueen’s bustard	660
A0A094L9Z6_PODCR *Serum albumin*	*Podiceps cristatus*	Great crested grebe	657
A0A093I422_STRCA *Serum albumin*	*Struthio camelus australis*	South African ostrich	648
A0A099ZYE0_CHAVO *Alpha-2-macroglobulin*	*Charadrius vociferus*	Killdeer	642
A0A0Q3TBH9_AMAAE *Fibronectin isoform X1*	*Amazona aestiva*	Turquoise-fronted parrot	631
A0A091WH83_NIPNI *Serum albumin*	*Nipponia nippon*	Japanese crested ibis	626
U3K0Q3_FICAL *Serum albumin*	*Ficedula albicollis*	Collared flycatcher	620
A0A099ZCF9_TINGU *Alpha-2-macroglobulin*	*Tinamus guttatus*	White-throated tinamou	551
A0A091PLB4_APAVI *Alpha-1-antiproteinase 2*	*Apaloderma vittatum*	Bar-tailed trogon	512
R7VRC4_COLLI *Complement C3*	*Columba livia*	Rock dove	448
A0A093B942_CHAPE *Apolipoprotein A-I*	*Chaetura pelagica*	Chimney swift	441
A0A093SYV6_PHACA *Ceruloplasmin*	*Phalacrocorax carbo*	Great cormorant	404
A0A087RBR7_APTFO *Ceruloplasmin*	*Aptenodytes forsteri*	Emperor penguin	401
P02118|HBB_ANSIN *Hemoglobin subunit beta*	*Anser indicus*	Bar-headed goose	392
A0A093GD58_DRYPU *Serum albumin*	*Dryobates pubescens*	Downy woodpecker	387
A0A493T9F7_ANAPP *Complement C3*	*Anas platyrhynchos platyrhynchos*	Mallard	349
A0A091KTR5_COLST *Alpha-2-macroglobulin*	*Colius striatus*	Speckled mousebird	348
A0A091P984_HALAL *Ovotransferrin*	*Haliaeetus albicilla*	White-tailed eagle	315
A0A091K9S4_COLST *Fibrinogen beta chain*	*Colius striatus*	Speckled mousebird	258
A0A091EDU9_CORBR *Alpha-1-antiproteinase 2*	*Corvus brachyrhynchos*	American crow	252
A0A094LH36_PODCR *Ovotransferrin*	*Podiceps cristatus*	Great crested grebe	252
A0A091LCI0_CATAU *Plasminogen*	*Cathartes aura*	Turkey vulture	240
A0A091SCH1_NESNO *Ovotransferrin*	*Nestor notabilis*	Kea	240
A0A091VG30_PHORB *Ceruloplasmin*	*Phoenicopterus ruber ruber*	American flamingo	229
P82111|HBA1_CATMA *Hemoglobin subunit alpha-1*	*Catharacta maccormicki*	South polar skua	227
A0A091UEL8_PHORB *Ovotransferrin*	*Phoenicopterus ruber ruber*	American flamingo	220
A0A093PT75_9PASS *Ovotransferrin*	*Manacus vitellinus*	Golden-collared manakin	218
A0A3L8SW70_CHLGU *Fibrinogen alpha chain*	*Chloebia gouldiae*	Gouldian finch	199
G1MPR2_MELGA *Complement C3*	*Meleagris gallopavo*	Wild turkey	194
A0A091J7H5_EGRGA *Ig heavy chain V region 5A*	*Egretta garzetta*	Little egret	188
S5MN40_ANTVP *Complement component 3d*	*Antigone vipio*	White-naped crane	182
A0A093Q6I9_9PASS *Ceruloplasmin*	*Manacus vitellinus*	Golden-collared manakin	158
A0A093KTV7_EURHL *Complement factor H*	*Eurypyga helias*	Sunbittern	153
A0A226NSR6_COLVI *Fibrinogen gamma chain*	*Colinus virginianus*	Northern bobwhite	148
A0A0A0AI07_CHAVO *Apolipoprotein B-100*	*Charadrius vociferus*	Killdeer	143
A0A091SMJ2_PELCR *Serum albumin*	*Pelecanus crispus*	Dalmatian pelican	130
A0A091P1L3_HALAL *Ig heavy chain V-III region GAL*	*Haliaeetus albicilla*	White-tailed eagle	130
A0A087REW6_APTFO *Glutathione peroxidase*	*Aptenodytes forsteri*	Emperor penguin	129
A0A093ELS8_TYTAL *Complement factor H*	*Tyto alba*	Barn owl	123
A0A091IHM8_CALAN *Complement factor H*	*Calypte anna*	Anna’s hummingbird	121
A0A087VMC1_BALRE *Alpha-1-antiproteinase*	*Balearica regulorum gibbericeps*	Grey crowned crane	120
A0A1V4JT28_PATFA *Fibrinogen alpha chain*	*Patagioenas fasciata monilis*	Band-tailed pigeon (western)	119
A0A087VMC3_BALRE *Alpha-1-antiproteinase 2*	*Balearica regulorum gibbericeps*	Grey crowned crane	113
A0A087QKE2_APTFO *Complement C1q subcomponent subunit A*	*Aptenodytes forsteri*	Emperor penguin	104
A0A091LYH7_CARIC *Complement receptor type 2*	*Cariama cristata*	Red-legged seriema	98
A0A3M0JM35_HIRRU *Histidine-rich glycoprotein*	*Hirundo rustica rustica*	Barn swallow	93
A0A091RP12_9GRUI *Selenoprotein P*	*Chlamydotis macqueenii*	MacQueen’s bustard	93
A0A087QZ39_APTFO *Retinol-binding protein 4*	*Aptenodytes forsteri*	Emperor penguin	90
A0A087QPM6_APTFO *Complement receptor type 2*	*Aptenodytes forsteri*	Emperor penguin	88
A0A2I0TTX4_LIMLA *Kininogen-1*	*Limosa lapponica baueri*	Bar-tailed godwit	87
A0A493T828_ANAPP *Complement C9*	*Anas platyrhynchos platyrhynchos*	Mallard	84
A0A093KM83_FULGA *Ovotransferrin*	*Fulmarus glacialis*	Northern fulmar	84
A0A091IQJ3_EGRGA *Ig heavy chain V-III region VH26*	*Egretta garzetta*	Little egret	66
A0A087VGQ5_BALRE *Ovotransferrin*	*Balearica regulorum gibbericeps*	Grey crowned crane	62
A0A2I0T8K5_LIMLA *Complement c3*	*Limosa lapponica baueri*	Bar-tailed godwit	58
A0A0A0ANE6_CHAVO *Ig heavy chain V-III region VH26*	*Charadrius vociferus*	Killdeer	56
A0A0A0APT8_CHAVO *Ig heavy chain V-III region HIL*	*Charadrius vociferus*	Killdeer	48
A0A0Q3U0C5_AMAAE *Alpha-tectorin-like protein*	*Amazona aestiva*	Turquoise-fronted parrot	44
Q9PRR6_9AVES *Apolipoprotein AI*	*Anser anser*	Greylag goose	44
A0A218V306_9PASE *Alpha-1-antiproteinase*	*Lonchura striata domestica*	Bengalese finch	41
A0A091TC37_PHALP *Ovoinhibitor*	*Phaethon lepturus*	White-tailed tropicbird	41

^ⱡ^ Ions score is −10. * Log (P), where P is the probability that the observed match is a random event. Individual ions scores > 22 indicated identity or extensive homology (*p* < 0.05). Protein scores were derived from ions scores as a non-probabilistic basis for ranking protein hits.

**Table 3:** Deiminated proteins identified by F95 enrichment in plasma of wandering albatross (*Diomedea exulans*). Deiminated proteins were isolated by immunoprecipitation using the pan-deimination F95 antibody. The F95-enriched eluate was analysed by LC–MS/MS and peak list files were submitted to mascot. Peptides matching with Aves_class_20190709 (876,224 sequences; 364,491,521 residues) are listed and total score is reported. Protein hits with Aves are indicated, including species name. Protein hits that were identified as deiminated in wandering albatross only, but not in northern giant petrel or south polar skua, are listed first and highlighted in pink and with an asterix (*). For full LC–MS/MS data analysis, see Supplementary Table S3.

Protein Name	Species Name	Common Name	Total Score (*p* < 0.05) ^ⱡ^
* A0A091VZN2_NIPNI *Uncharacterized protein*	*Nipponia nippon*	Japanese crested ibis	942
* A0A093HL59_STRCA *Uncharacterized protein*	*Struthio camelus australis*	South African ostrich	658
* A0A493T350_ANAPP *Uncharacterized protein*	*Anas platyrhynchos platyrhynchos*	Mallard	388
* A0A091LY76_CATAU *Deleted in malignant brain tumours 1 protein*	*Cathartes aura*	Turkey vulture	299
* A0A2I0TFB3_LIMLA *Soluble scavenger receptor cysteine-rich domain-containing protein ssc5d-like*	*Limosa lapponica baueri*	Bar-tailed godwit	245
* A0A160F7C0_TAEGU *Corticosteroid binding globulin*	*Taeniopygia guttata*	Zebra finch	226
* A0A226MDB4_CALSU *Uncharacterized protein*	*Callipepla squamata*	Scaled quail	203
* A0A3Q3B296_CHICK *Uncharacterized protein*	*Gallus gallus*	Chicken	187
* A0A3L8SF82_CHLGU *Uncharacterized protein*	*Chloebia gouldiae*	Gouldian finch	181
* A0A068L966_STRCA *Beta-actin*	*Struthio camelus australis*	South African ostrich	138
* A0A0Q3MUK2_AMAAE *Uncharacterized protein*	*Amazona aestiva*	turquoise-fronted parrot	127
* A0A087QIW1_APTFO *Ig lambda chain V-1 region*	*Aptenodytes forsteri*	Emperor penguin	117
* A0A0Q3PU08_AMAAE *Ig gamma-1 chain C region, membrane-bound form*	*Amazona aestiva*	Turquoise-fronted parrot	100
* A0A091W8Q2_OPIHO *Vitamin D-binding protein*	*Opisthocomus hoazin*	Hoatzin (skunk bird, Canje pheasant)	92
* A0A3M0L7R0_HIRRU *Uncharacterized protein*	*Hirundo rustica rustica*	Barn swallow	68
* A0A226N4C8_CALSU *Apolipoprotein AIV*	*Callipepla squamata*	Scaled quail	68
* A0A087QM54_APTFO *Complement C4*	*Aptenodytes forsteri*	Emperor penguin	64
* A0A087QZU5_APTFO *Vitronectin*	*Aptenodytes forsteri*	Emperor penguin	62
* A0A1V4KQ91_PATFA *Lipid phosphate phosphatase-related protein type 3-like*	*Patagioenas fasciata monilis*	Band-tailed pigeon (western)	60
* A0A087QZ39_APTFO *Retinol-binding protein 4*	*Aptenodytes forsteri*	Emperor penguin	54
* A0A493T0F4_ANAPP *Uncharacterized protein*	*Anas platyrhynchos platyrhynchos*	Mallard	53
* A0A493U126_ANAPP *Uncharacterized protein*	*Anas platyrhynchos platyrhynchos*	Mallard	51
* A0A091GEI4_9AVES *Ubiquitin carboxyl-terminal hydrolase*	*Cuculus canorus*	Common cuckoo	47
* A0A087R4Q6_APTFO *Noelin*	*Aptenodytes forsteri*	Emperor penguin	47
* A0A091ECG6_CORBR *Coiled-coil domain-containing protein 112*	*Corvus brachyrhynchos*	American crow	45
* A0A099ZM42_TINGU *Collagen alpha-4 (VI) chain*	*Tinamus guttatus*	White-throated tinamou	45
* A0A3L8SDK7_CHLGU *Outer dense fiber protein 2*	*Chloebia gouldiae*	Gouldian finch	45
* A0A094K563_ANTCR *SET and MYND domain-containing protein 4*	*Antrostomus carolinensis*	Chuck-will’s-widow	44
A0A093P0F9_PYGAD *Serum albumin*	*Pygoscelis adeliae*	Adélie penguin	1696
A0A093FHI9_GAVST *Serum albumin*	*Gavia stellata*	Red-throated loon	1587
A0A087R4G9_APTFO *Alpha-2-macroglobulin*	*Aptenodytes forsteri*	Emperor penguin	1400
A0A093F817_TYTAL *Serum albumin*	*Tyto alba*	Barn owl	1376
A0A091UPZ3_PHALP *Serum albumin*	*Phaethon lepturus*	White-tailed tropicbird	1180
A0A0Q3X9Z0_AMAAE *Serum albumin-like protein*	*Amazona aestiva*	Turquoise-fronted parrot	1163
A0A0Q3PZX3_AMAAE *Fibrinogen*	*Amazona aestiva*	Turquoise-fronted parrot	1162
A0A094KA73_ANTCR *Beta-fibrinogen*	*Antrostomus carolinensis*	Chuck-will’s-widow	1068
A0A0A0A1J2_CHAVO *Alpha-2-macroglobulin*	*Charadrius vociferus*	Killdeer	1033
A0A094L652_ANTCR *Serum albumin*	*Antrostomus carolinensis*	Chuck-will’s-widow	1004
A0A091LFY3_9GRUI *Fibrinogen*	*Chlamydotis macqueenii*	MacQueen’s bustard	961
A0A093KX01_FULGA *Alpha-2-macroglobulin*	*Fulmarus glacialis*	Northern fulmar	957
A0A087VH79_BALRE *Fibrinogen*	*Balearica regulorum gibbericeps*	Grey crowned crane	923
R0M0W6_ANAPL *Serum albumin*	*Anas platyrhynchos*	Mallard	873
A0A087RBR7_APTFO *Ceruloplasmin*	*Aptenodytes forsteri*	Emperor penguin	828
A0A087VA40_BALRE *Fibronectin*	*Balearica regulorum gibbericeps*	Grey crowned crane	820
A0A1V4JT04_PATFA *Fibrinogen gamma chain*	*Patagioenas fasciata monilis*	Band-tailed pigeon (western)	759
A0A099ZCF9_TINGU *Alpha-2-macroglobulin*	*Tinamus guttatus*	White-throated tinamou	748
A0A091SGY4_PELCR *Ceruloplasmin*	*Pelecanus crispus*	Dalmatian pelican	747
A0A0A0A3R1_CHAVO *Apolipoprotein A-I*	*Charadrius vociferus*	Killdeer	737
P19121|ALBU_CHICK *Serum albumin*	*Gallus gallus*	Chicken	727
A0A093GBQ7_DRYPU *Fibronectin*	*Dryobates pubescens*	Downy woodpecker	635
A0A093INM3_FULGA *Fibrinogen alpha chain*	*Fulmarus glacialis*	Northern fulmar	634
A0A2I0UMY8_LIMLA *Fibrinogen gamma chain*	*Limosa lapponica baueri*	Bar-tailed godwit	628
A0A093PBF1_PYGAD *Alpha-2-macroglobulin*	*Pygoscelis adeliae*	Adélie penguin	599
A0A093FGC0_GAVST *Fibrinogen alpha chain*	*Gavia stellata*	Red-throated loon	589
A0A093G3Z1_DRYPU *Fibrinogen alpha chain*	*Dryobates pubescens*	Downy woodpecker	588
A0A0Q3LVM5_AMAAE *Apolipoprotein A-I*	*Amazona aestiva*	turquoise-fronted parrot	551
A0A087RJ23_APTFO *Kininogen-1*	*Aptenodytes forsteri*	Emperor penguin	523
A0A2I0TGV4_LIMLA *Serum albumin*	*Limosa lapponica baueri*	Bar-tailed godwit	521
O42296|APOA1_ANAPL *Apolipoprotein A-I*	*Anas platyrhynchos*	Mallard	515
A0A093QN86_9PASS *Serum albumin*	*Manacus vitellinus*	Golden-collared manakin	492
A0A091SMJ2_PELCR *Serum albumin*	*Pelecanus crispus*	Dalmatian pelican	460
A0A0Q3US23_AMAAE *Kininogen-1*	*Amazona aestiva*	turquoise-fronted parrot	438
A0A091VCC2_NIPNI *Apolipoprotein A-I*	*Nipponia nippon*	Japanese crested ibis	431
A0A087R543_APTFO *Alpha-1-antiproteinase 2*	*Aptenodytes forsteri*	Emperor penguin	399
A0A099ZYE0_CHAVO *Alpha-2-macroglobulin*	*Charadrius vociferus*	Killdeer	382
A0A093BVV9_TAUER *Kininogen-1*	*Tauraco erythrolophus*	Red-crested turaco	377
A0A3M0KRB0_HIRRU *Fibrinogen*	*Hirundo rustica rustica*	Barn swallow	374
A0A091EST7_CORBR *Alpha-2-macroglobulin*	*Corvus brachyrhynchos*	American crow	352
A0A093BMK0_9AVES *Ovotransferrin*	*Pterocles gutturalis*	Yellow-throated sandgrouse	349
A0A093CUQ3_9AVES *Fibrinogen alpha chain*	*Pterocles gutturalis*	Yellow-throated sandgrouse	346
A0A091LXC5_CARIC *Alpha-2-macroglobulin*	*Cariama cristata*	Red-legged seriema	336
A0A087VCN6_BALRE *Alpha-1-antiproteinase 2*	*Balearica regulorum gibbericeps*	Grey crowned crane	326
A0A087R9I5_APTFO *Complement factor H*	*Aptenodytes forsteri*	Emperor penguin	302
A0A087RBW2_APTFO *IgGFc-binding protein*	*Aptenodytes forsteri*	Emperor penguin	288
A0A093Q6I9_9PASS *Ceruloplasmin*	*Manacus vitellinus*	Golden-collared manakin	285
A0A093NV14_PYGAD *Complement factor H*	*Pygoscelis adeliae*	Adélie penguin	269
R7VRC4_COLLI *Complement C3*	*Columba livia*	Rock dove	259
A0A0A0AI70_CHAVO *Ovotransferrin*	*Charadrius vociferus*	Killdeer	238
A0A1D5P6F4_CHICK *IgGFc-binding protein*	*Gallus gallus*	Chicken	230
A0A0Q3U0C5_AMAAE *Alpha-tectorin-like protein*	*Amazona aestiva*	Turquoise-fronted parrot	226
A0A091P984_HALAL *Ovotransferrin*	*Haliaeetus albicilla*	White-tailed eagle	224
A0A087RBW1_APTFO *IgGFc-binding protein*	*Aptenodytes forsteri*	Emperor penguin	198
A0A091P1L3_HALAL *Ig heavy chain V-III region GAL*	*Haliaeetus albicilla*	White-tailed eagle	195
A0A087REW6_APTFO *Glutathione peroxidase*	*Aptenodytes forsteri*	Emperor penguin	186
A0A087QH18_APTFO *Plasminogen*	*Aptenodytes forsteri*	Emperor penguin	176
A0A093IJM0_FULGA *IgGFc-binding protein*	*Fulmarus glacialis*	Northern fulmar	174
A0A093GZX5_GAVST *Ovotransferrin*	*Gavia stellata*	Red-throated loon	173
A0A091V0T3_NIPNI *IgGFc-binding protein*	*Nipponia nippon*	Japanese crested ibis	169
A0A091GDA6_9AVES *Keratin, type I cytoskeletal 42*	*Cuculus canorus*	Common cuckoo	155
A0A091KHK5_9GRUI *IgGFc-binding protein*	*Chlamydotis macqueenii*	MacQueen’s bustard	155
A0A2I0LGF9_COLLI *Alpha-2-macroglobulin-like*	*Columba livia*	Rock dove	153
A0A493T9F7_ANAPP *Complement C3*	*Anas platyrhynchos platyrhynchos*	Mallard	150
A0A091SZR3_PELCR *Ig heavy chain V region C3*	*Pelecanus crispus*	Dalmatian pelican	147
A0A1V4KDF4_PATFA *Complement C1q tumor necrosis factor-related protein 3 isoform A*	*Patagioenas fasciata monilis*	Band-tailed pigeon (western)	143
A0A093ISV2_FULGA *IgGFc-binding protein*	*Fulmarus glacialis*	Northern fulmar	136
A0A091W577_NIPNI *IgGFc-binding protein*	*Nipponia nippon*	Japanese crested ibis	128
A0A087V679_BALRE *Selenoprotein P*	*Balearica regulorum gibbericeps*	Grey crowned crane	111
A0A087R546_APTFO *Alpha-1-antiproteinase 2*	*Aptenodytes forsteri*	Emperor penguin	111
A0A091HFG6_BUCRH *Complement factor H*	*Buceros rhinoceros silvestris*	Rhinoceros hornbill	102
R0L2Q3_ANAPL *IgGFc-binding protein*	*Anas platyrhynchos*	Mallard	94
A0A093CFV7_9AVES *Ig heavy chain V-III region CAM*	*Pterocles gutturalis*	Yellow-throated sandgrouse	74
I6UVI9_STRCA *Immunonoglobulin heavy chain variable region*	*Struthio camelus australis*	South African ostrich	56
A0A087R544_APTFO *Alpha-1-antiproteinase 2*	*Aptenodytes forsteri*	Emperor penguin	52
A0A226NM49_CALSU *Uncharacterized protein*	*Callipepla squamata*	Scaled quail	52
A0A091EVY3_CORBR *Ig heavy chain V region C3*	*Corvus brachyrhynchos*	American crow	47
A0A2I0TNP2_LIMLA *Selenoprotein pb-like*	*Limosa lapponica baueri*	Bar-tailed godwit	47
A0A091S5G4_NESNO *Complement C1q subcomponent subunit C*	*Nestor notabilis*	Kea	45
A0A093HG08_GAVST *Complement C1q subcomponent subunit A*	*Gavia stellata*	Red-throated loon	44

^ⱡ^ Ions score is −10. * Log (P), where P is the probability that the observed match is a random event. Individual ions scores > 22 indicated identity or extensive homology (*p* < 0.05). Protein scores were derived from ions scores as a non-probabilistic basis for ranking protein hits.

## Supplementary Materials

The following are available online at https://www.mdpi.com/2079-7737/9/1/15/s1. Table S1: LC–MS/MS analysis of F95-enriched protein hits identified in plasma of northern giant petrel (*Macronectes halli*). Deiminated proteins were isolated by immunoprecipitation using the pan-deimination F95 antibody. The F95-enriched eluate was analysed by LC–MS/MS and peak list files were submitted to mascot. Peptides matching with Aves_class_20190709 (876,224 sequences; 364,491,521 residues) are shown. Peptide sequences for individual protein hits, their m/z values and individual scores are listed. Table S2: LC–MS/MS analysis of F95-enriched protein hits identified in plasma of south polar skua (*Stercorarius maccormicki*). Deiminated proteins were isolated by immunoprecipitation using the pan-deimination F95 antibody. The F95-enriched eluate was analysed by LC–MS/MS and peak list files were submitted to mascot. Peptides matching with Aves_class_20190709 (876,224 sequences; 364,491,521 residues) are shown. Peptide sequences for individual protein hits, their m/z values and individual scores are listed. Table S3: LC–MS/MS analysis of F95-enriched protein hits identified in plasma of wandering albatross (*Diomedea exulans*). Deiminated proteins were isolated by immunoprecipitation using the pan-deimination F95 antibody. The F95-enriched eluate was analysed by LC–MS/MS and peak list files were submitted to mascot. Peptides matching with Aves_class_20190709 (876,224 sequences; 364,491,521 residues) are shown. Peptide sequences for individual protein hits, their m/z values and individual scores are listed.

## References

[1] Vossenaar E.R., Zendman A.J., van Venrooij W.J., Pruijn G.J. (2003). PAD, a Growing Family of Citrullinating Enzymes: Genes, Features and Involvement in Disease. Bioessays.

[2] György B., Toth E., Tarcsa E., Falus A., Buzas E.I. (2006). Citrullination: A Posttranslational Modification in Health and Disease. Int. J. Biochem. Cell Biol..

[3] Bicker K.L., Thompson P.R. (2013). The Protein Arginine Deiminases: Structure, Function, Inhibition, and Disease. Biopolymers.

[4] Wang S., Wang Y. (2013). Peptidylarginine Deiminases in Citrullination, Gene Regulation, Health and Pathogenesis. Biochim. Biophys. Acta.

[5] Lange S., Gallagher M., Kholia S., Kosgodage U.S., Hristova M., Hardy J., Inal J.M. (2017). Peptidylarginine Deiminases-Roles in Cancer and Neurodegeneration and Possible Avenues for Therapeutic Intervention via Modulation of Exosome and Microvesicle (EMV) Release?. Int. J. Mol. Sci..

[6] Magnadottir B., Hayes P., Hristova M., Bragason B.Þ., Nicholas A.P., Dodds A.W., Gudmundsdottir S., Lange S. (2018). Post-translational Protein Deimination in Cod (*Gadus morhua* L.) Ontogeny-Novel Roles in Tissue Remodelling and Mucosal Immune Defences?. Dev. Comp. Immunol..

[7] Magnadottir B., Bragason B.T., Bricknell I.R., Bowden T., Nicholas A.P., Hristova M., Gudmundsdottir S., Dodds A.W., Lange S. (2019). Peptidylarginine Deiminase and Deiminated Proteins are detected throughout Early Halibut Ontogeny-Complement Components C3 and C4 are Post-Translationally Deiminated in Halibut (*Hippoglossus hippoglossus* L.). Dev. Comp. Immunol..

[8] Magnadottir B., Kraev I., Guðmundsdóttir S., Dodds A.W., Lange S. (2019). Extracellular Vesicles from Cod (*Gadus morhua* L.) Mucus Contain Innate Immune Factors and Deiminated Protein Cargo. Dev. Comp. Immunol..

[9] Criscitiello M.F., Kraev I., Lange S. (2019). Deiminated Proteins in Extracellular Vesicles and Plasma of Nurse Shark (*Ginglymostoma cirratum*)-Novel Insights into Shark Immunity. Fish Shellfish Immunol..

[10] Criscitiello M.F., Kraev I., Lange S. (2020). Deiminated Proteins in Extracellular Vesicles and Serum of Llama (*Lama glama*)-Novel Insights into Camelid Immunity. Mol. Immunol..

[11] Pamenter M.E., Uysal-Onganer P., Huynh K.W., Kraev I., Lange S. (2019). Post-translational Deimination of Immunological and Metabolic Protein Markers in Plasma and Extracellular Vesicles of Naked Mole-Rat (*Heterocephalus glaber*). Int. J. Mol. Sci..

[12] Witalison E.E., Thompson P.R., Hofseth L.J. (2015). Protein Arginine Deiminases and Associated Citrullination: Physiological Functions and Diseases Associated with Dysregulation. Curr. Drug Targets.

[13] Henderson B., Martin A.C. (2014). Protein Moonlighting: A New Factor in Biology and Medicine. Biochem. Soc. Trans..

[14] Jeffrey C.J. (2018). Protein Moonlighting: What is it, and Why is it Important?. Philos. Trans. R. Soc. Lond. B Biol. Sci..

[15] Rebl A., Köllner B., Anders E., Wimmers K., Goldammer T. (2010). Peptidylarginine Deiminase Gene is Differentially Expressed in Freshwater and Brackish Water Rainbow Trout. Mol. Biol. Rep..

[16] Lange S., Gögel S., Leung K.Y., Vernay B., Nicholas A.P., Causey C.P., Thompson P.R., Greene N.D., Ferretti P. (2011). Protein Deiminases: New Players in the Developmentally Regulated Loss of Neural Regenerative Ability. Dev. Biol..

[17] Bielecka E., Scavenius C., Kantyka T., Jusko M., Mizgalska D., Szmigielski B., Potempa B., Enghild J.J., Prossnitz E.R., Blom A.M. (2014). Peptidyl Arginine Deiminase from *Porphyromonas gingivalis* Abolishes Anaphylatoxin C5a Activity. J. Biol. Chem..

[18] Kosgodage U.S., Matewele P., Mastroianni G., Kraev I., Brotherton D., Awamaria B., Nicholas A.P., Lange S., Inal J.M. (2019). Peptidylarginine Deiminase Inhibitors Reduce Bacterial Membrane Vesicle Release and Sensitize Bacteria to Antibiotic Treatment. Front. Cell. Infect. Microbiol..

[19] Gavinho B., Rossi I.V., Evans-Osses I., Lange S., Ramirez M.I. (2019). Peptidylarginine Deiminase Inhibition Abolishes the Production of Large Extracellular Vesicles from *Giardia intestinalis*, Affecting Host-Pathogen Interactions by Hindering Adhesion to Host Cells. bioRxiv.

[20] El-Sayed A.S.A., Shindia A.A., AbouZaid A.A., Yassin A.M., Ali G.S., Sitohy M.Z. (2019). Biochemical Characterization of Peptidylarginine Deiminase-Like Orthologs from Thermotolerant *Emericella Dentata* and Aspergillus Nidulans. Enzyme Microb. Technol..

[21] Lange S., Rocha-Ferreira E., Thei L., Mawjee P., Bennett K., Thompson P.R., Subramanian V., Nicholas A.P., Peebles D., Hristova M. (2014). Peptidylarginine Deiminases: Novel Drug Targets for Prevention of Neuronal Damage following Hypoxic Ischemic Insult (HI) in Neonates. J. Neurochem..

[22] Lange S. (2016). Peptidylarginine Deiminases as Drug Targets in Neonatal Hypoxic-Ischemic Encephalopathy. Front. Neurol..

[23] Magnadottir B., Hayes P., Gísladóttir B., Bragason B., Hristova M., Nicholas A.P., Guðmundsdóttir S., Lange S. (2018). Pentraxins CRP-I and CRP-II are Post-Translationally Deiminated and Differ in Tissue Specificity in Cod (*Gadus morhua* L.) Ontogeny. Dev. Comp. Immunol..

[24] Magnadottir B., Uysal-Onganer P., Kraev I., Svansson V., Lange S. (2020). Deiminated Proteins and Extracellular Vesicles-Novel Serum Biomarkers in Whales and Orca. Comp. Biochem. Physiol. Part D.

[25] Kholia S., Jorfi S., Thompson P.R., Causey C.P., Nicholas A.P., Inal J.M., Lange S. (2015). A Novel Role for Peptidylarginine Deiminases in Microvesicle Release Reveals Therapeutic potential of PAD Inhibition in Sensitizing Prostate Cancer Cells to Chemotherapy. J. Extracell. Vesicles.

[26] Kosgodage U.S., Trindade R.P., Thompson P.R., Inal J.M., Lange S. (2017). Chloramidine/Bisindolylmaleimide-I-Mediated Inhibition of Exosome and Microvesicle Release and Enhanced Efficacy of Cancer Chemotherapy. Int. J. Mol. Sci..

[27] Kosgodage U.S., Onganer P.U., Maclatchy A., Nicholas A.P., Inal J.M., Lange S. (2018). Peptidylarginine Deiminases Post-translationally Deiminate Prohibitin and Modulate Extracellular Vesicle Release and miRNAs 21 and 126 in Glioblastoma Multiforme. Int. J. Mol. Sci..

[28] Inal J.M., Ansa-Addo E.A., Lange S. (2013). Interplay of Host-Pathogen Microvesicles and Their Role in Infectious Disease. Biochem. Soc. Trans..

[29] Colombo M., Raposo G., Théry C. (2014). Biogenesis, Secretion, and Intercellular Interactions of Exosomes and Other Extracellular Vesicles. Annu. Rev. Cell Dev. Biol..

[30] Turchinovich A., Drapkina O., Tonevitsky A. (2019). Transcriptome of Extracellular Vesicles: State-of-the-Art. Front. Immunol..

[31] Vagner T., Chin A., Mariscal J., Bannykh S., Engman D.M., Di Vizio D. (2019). Protein Composition Reflects Extracellular Vesicle Heterogeneity. Proteomics.

[32] Hessvik N.P., Llorente A. (2018). Current knowledge on Exosome Biogenesis and Release. Cell Mol. Life Sci..

[33] Ramirez S.H., Andrews A.M., Paul D., Pachter J.S. (2018). Extracellular Vesicles: Mediators and Biomarkers of Pathology along CNS Barriers. Fluids Barriers CNS.

[34] Iliev D., Strandskog G., Nepal A., Aspar A., Olsen R., Jørgensen J., Wolfson D., Ahluwalia B.S., Handzhiyski J., Mironova R. (2018). Stimulation of Exosome Release by Extracellular DNA is Conserved Across Multiple Cell Types. FEBS J..

[35] Lange S., Kraev I., Magnadóttir B., Dodds A.W. (2019). Complement Component C4-Like Protein in Atlantic Cod (*Gadus morhua* L.)-Detection in Ontogeny and Identification of Post-Translational Deimination in Serum and Extracellular Vesicles. Dev. Comp. Immunol..

[36] Sun Y., Saito K., Saito Y. (2019). Lipid Profile Characterization and Lipoprotein Comparison of Extracellular Vesicles from Human Plasma and Serum. Metabolites.

[37] Kosgodage U.S., Matewele P., Awamaria B., Kraev I., Warde P., Mastroianni G., Nunn A.V., Guy G.W., Bell J.D., Inal J.M. (2019). Cannabidiol Is a Novel Modulator of Bacterial Membrane Vesicles. Front. Cell Infect. Microbiol..

[38] Magnadottir B., Uysal-Onganer P., Kraev I., Dodds A.W., Gudmundsdottir S., Lange S. (2020). Extracellular Vesicles, Deiminated Protein Cargo and microRNAs are Novel Serum Biomarkers for Environmental Rearing Temperature in Atlantic cod (*Gadus morhua* L.). Aquac. Rep..

[39] Anderson O.R.J., Phillips R.A., McDonald R.A., Shore R.F., McGill R.A.R., Bearhop S. (2009). Influence of Trophic Position and Foraging Range on Mercury Levels within a Seabird Community. Mar. Ecol. Prog. Ser..

[40] Phillips R.A., Gales R., Baker G.B., Double M.C., Favero M., Quintana F., Tasker M.L., Weimerskirch H., Uhart M., Wolfaardt A. (2016). The Conservation Status and Priorities for Albatrosses and Large Petrels. Biol. Conserv..

[41] Dias M.P., Martin R., Pearmain E.J., Burfield I.J., Small C., Phillips R.A., Yates O., Lascelles B., Borboroglu P.G., Croxall J.P. (2019). Threats to Seabirds: A Global Assessment. Biol. Conserv..

[42] Barbraud C., Rivalan P., Inchausti P., Nevoux M., Rolland V., Weimerskirch H. (2011). Contrasted Demographic Responses Facing Future Climate Change in Southern Ocean Seabirds. J. Anim. Ecol..

[43] Grecian W.J., Taylor G.A., Loh G., McGill R.A.R., Miskelly C.M., Phillips R.A., Thompson D.R., Furness R.W. (2016). Contrasting Migratory Responses of Two Closely-Related Seabirds to Long-Term Climate Change. Mar. Ecol. Prog. Ser..

[44] Pardo D., Forcada J., Wood A.G., Tuck G.N., Ireland L., Pradel R., Croxall J.P., Phillips R.A. (2017). Additive Effects of Climate and Fisheries Drive Ongoing Declines in Multiple Albatross species. Proc. Natl. Acad. Sci. USA.

[45] Anderson O.R.J., Phillips R.A., Shore R.F., McGill R.A.R., McDonald R.A., Bearhop S. (2010). Element Patterns in Albatrosses and Petrels: Influence of Trophic Position, Foraging Range, and Prey Type. Environ. Pollut..

[46] Leat E.H.K., Bourgeon S., Magnusdottir E., Gabrielsen G.W., Grecian W.J., Hanssen S.A., Olafsdottir K., Petersen A., Phillips R.A., Strøm H. (2013). The Influence of Wintering Area on Concentration and Pattern of Persistent Organic Pollutants in a Breeding Migratory Seabird. Mar. Ecol. Prog. Ser..

[47] Cherel Y., Barbraud C., Lahournat M., Jaeger A., Jaquemet S., Wanless R.M., Phillips R.A., Thompson D.R., Bustamante P. (2018). Accumulate or Eliminate? Seasonal Mercury Dynamics in Albatrosses, the Most Contaminated Family of Birds. Environ. Pollut..

[48] Uhart M.M., Gallo L., Quintana F. (2018). Review of Diseases (Pathogen Isolation, Direct Recovery and Antibodies) in Albatrosses and Large Petrels Worldwide. Bird Conserv. Int..

[49] Leotta G.A., Rivas M., Chinen I., Vigo G.B., Moredo F.A., Coria N., Wolcott M.J. (2003). Avian Cholera in a Southern Giant Petrel (Macronectes Giganteus) from Antarctica. J. Wildl. Dis..

[50] Descamps S., Jenouvrier S., Gilchrist H.G., Forbes M.R. (2012). Avian Cholera, a Threat to the Viability of an Arctic Seabird Colony?. PLoS ONE.

[51] Jaeger A., Lebarbenchon C., Bourret V., Bastien M., Lagadec E., Thiebot J.B., Boulinier T., Delord K., Barbraud C., Marteau C. (2018). Avian Cholera Outbreaks Threaten Seabird Species on Amsterdam Island. PLoS ONE.

[52] Gamble A., Garnier R., Jaeger A., Gantelet H., Thibault E., Tortosa P., Bourret V., Thiebot J.B., Delord K., Weimerskirch H. (2019). Exposure of Breeding Albatrosses to the Agent of Avian Cholera: Dynamics of Antibody Levels and Ecological Implications. Oecologia.

[53] Tompkins E.M., Anderson D.J., Pabilonia K.L., Huyvaert K.P. (2017). Avian Pox Discovered in the Critically Endangered Waved Albatross. J. Wildl. Dis..

[54] Wilkinson D.A., Dietrich M., Lebarbenchon C., Jaeger A., Le Rouzic C., Bastien M., Lagadec E., McCoy K.D., Pascalis H., Le Corre M. (2014). Massive Infection of Seabird Ticks with Bacterial Species Related to Coxiella burnetii. Appl. Environ. Microbiol..

[55] Arnal A., Vittecoq M., Pearce-Duvet J., Gauthier-Clerc M., Boulinier T., Jourdain E. (2015). Laridae: A neglected Reservoir that could Play a Major Role in Avian Influenza Virus Epidemiological Dynamics. Crit. Rev. Microbiol..

[56] Jaeger A., Lecollinet S., Beck C., Bastien M., Le Corre M., Dellagi K., Pascalis H., Boulinier T., Lebarbenchon C. (2016). Serological Evidence for the Circulation of Flaviviruses in Seabird Populations of the Western Indian Ocean. Epidemiol. Infect..

[57] Dupraz M., Toty C., Devillers E., Blanchon T., Elguero E., Vittecoq M., Moutailler S., McCoy K.D. (2017). Population Structure of the Soft Tick *Ornithodoros maritimus* and its Associated Infectious Agents within a Colony of its Seabird Host Larus Michahellis. Int. J. Parasitol. Parasites Wildl..

[58] Ayadi T., Selmi S., Hammouda A., Reis S., Boulinier T., Loiseau C. (2018). Diversity, Prevalence and Host Specificity of Avian Parasites in Southern Tunisian Oases. Parasitology.

[59] Gamble A., Ramos R., Parra-Torres Y., Mercier A., Galal L., Pearce-Duvet J., Villena I., Montalvo T., González-Solís J., Hammouda A. (2019). Exposure of Yellow-Legged gulls to Toxoplasma Gondii along the Western Mediterranean Coasts: Tales from a Sentinel. Int. J. Parasitol. Parasites Wildl..

[60] Khan J.S., Provencher J.F., Forbes M.R., Mallory M.L., Lebarbenchon C., McCoy K.D. (2019). Parasites of Seabirds: A Survey of Effects and Ecological Implications. Adv. Mar. Biol..

[61] Sanz-Aguilar A., Payo-Payo A., Rotger A., Yousfi L., Moutailler S., Beck C., Dumarest M., Igual J.M., Miranda M.Á., Viñas Torres M. (2019). Infestation of small seabirds by Ornithodoros maritimus Ticks: Effects on Chick Body Condition, Reproduction and Associated Infectious agents. Ticks Tick Borne Dis..

[62] Finkelstein M., Grasman K.A., Croll D.A., Tershy B., Smith D.R. (2003). Immune Function of Cryopreserved Avian Peripheral White Blood Cells: Potential Biomarkers of Contaminant Effects in Wild Birds. Arch. Environ. Contam. Toxicol..

[63] Finkelstein M.E., Grasman K.A., Croll D.A., Tershy B.R., Keitt B.S., Jarman W.M., Smith D.R. (2007). Contaminant-Associated Alteration of Immune Function in Black-Footed Albatross (*Phoebastria nigripes*), a North Pacific Predator. Environ. Toxicol. Chem..

[64] Bourgeon S., Leat E.H., Magnusdóttir E., Fisk A.T., Furness R.W., Strøm H., Hanssen S.A., Petersen A., Olafsdóttir K., Borgå K. (2012). Individual Variation in Biomarkers of Health: Influence of Persistent Organic Pollutants in Great Skuas (*Stercorarius skua*) Breeding at Different Geographical Locations. Environ. Res..

[65] Provencher J.F., Forbes M.R., Hennin H.L., Love O.P., Braune B.M., Mallory M.L., Gilchrist H.G. (2016). Implications of Mercury and Lead Concentrations on Breeding Physiology and Phenology in an Arctic Bird. Environ. Pollut..

[66] Sebastiano M., Eens M., Angelier F., Pineau K., Chastel O., Costantini D. (2017). Corticosterone, Inflammation, Immune Status and Telomere Length in Frigatebird Nestlings Facing a Severe Herpesvirus Infection. Conserv. Physiol..

[67] O’Reilly E.L., Eckersall P.D. (2014). Acute Phase Proteins: A Review of Their Function, Behaviour and Measurement in Chickens. Worlds Poult. Sci. J..

[68] Zulkifli I., Najafi P., Nurfarahin A.J., Soleimani A.F., Kumari S., Aryani A.A., O’Reilly E.L., Eckersall P.D. (2014). Acute Phase Proteins, Interleukin 6, and Heat Shock Protein 70 in Broiler Chickens Administered with Corticosterone. Poult. Sci..

[69] Horvatić A., Guillemin N., Kaab H., McKeegan D., O’Reilly E., Bain M., Kuleš J., Eckersall P.D. (2018). Integrated Dataset on Acute Phase Protein Response in Chicken Challenged with *Escherichia coli* Lipopolysaccharide Endotoxin. Data Brief.

[70] O’Reilly E.L., Bailey R.A., Eckersall P.D. (2018). A Comparative Study of Acute-Phase Protein Concentrations in Historical and Modern Broiler Breeding Lines. Poult. Sci..

[71] Horvatić A., Guillemin N., Kaab H., McKeegan D., O’Reilly E., Bain M., Kuleš J., Eckersall P.D. (2019). Quantitative Proteomics Using Tandem Mass Tags in Relation to the Acute Phase Protein Response in Chicken Challenged with *Escherichia coli* Lipopolysaccharide Endotoxin. J. Proteom..

[72] Théry C., Witwer K.W., Aikawa E., Alcaraz M.J., Anderson J.D., Andriantsitohaina R., Antoniou A., Arab T., Archer F., Atkin-Smith G.K. (2018). Minimal Information for Studies of Extracellular Vesicles 2018 (MISEV2018): A Position Statement of the International Society for Extracellular Vesicles and update of the MISEV2014 guidelines. J. Extracell Vesicles.

[73] Soo C.Y., Song Y., Zheng Y., Campbell E.C., Riches A.C., Gunn-Moore F., Zheng Y., Powis S.J. (2012). Nanoparticle Tracking Analysis Monitors Microvesicle and Exosome Secretion from Immune Cells. Immunology.

[74] Nicholas A.P., Whitaker J.N. (2002). Preparation of a Monoclonal Antibody to Citrullinated Epitopes: Its Characterization and Some Applications to Immunohistochemistry in Human Brain. Glia.

[75] Peters T. (1996). All about Albumin. Biochemistry, Genetics, and Medical Applications.

[76] White C.R., Datta G., Giordano S. (2017). High-Density Lipoprotein Regulation of Mitochondrial Function. Adv. Exp. Med. Biol..

[77] Arciello A., Piccoli R., Monti D.M. (2016). Apolipoprotein A-I: The Dual Face of a Protein. FEBS Lett..

[78] Jenne D.E., Lowin B., Peitsch M.C., Böttcher A., Schmitz G., Tschopp J. (1991). Clusterin (Complement Lysis Inhibitor) Forms a High Density Lipoprotein Complex with Apolipoprotein A-I in Human Plasma. J. Biol. Chem..

[79] Hamilton K.K., Zhao J., Sims P.J. (1993). Interaction between Apolipoproteins A-I and A-II and the Membrane Attack Complex of Complement. Affinity of the Apoproteins for Polymeric C9. J. Biol. Chem..

[80] Magnadottir B., Lange S. (2004). Is Apolipoprotein A-I a Regulating Protein for the Complement System of Cod (*Gadus morhua* L.)?. Fish Shellfish Immunol..

[81] Qu J., Ko C.W., Tso P., Bhargava A. (2019). Apolipoprotein A-IV: A Multifunctional Protein Involved in Protection against Atherosclerosis and Diabetes. Cells.

[82] Peterson M.M., Mack J.L., Hall P.R., Alsup A.A., Alexander S.M., Sully E.K., Sawires Y.S., Cheung A.L., Otto M., Gresham H.D. (2008). Apolipoprotein B is an Innate Barrier Against Invasive Staphylococcus Aureus Infection. Cell Host Microbe.

[83] Su Q., Tsai J., Xu E., Qiu W., Bereczki E., Santha M., Adeli K. (2009). Apolipoprotein B100 Acts as a Molecular Link between Lipid-Induced Endoplasmic Reticulum Stress and Hepatic Insulin Resistance. Hepatology.

[84] Andersen L.H., Miserez A.R., Ahmad Z., Andersen R.L. (2016). Familial Defective Apolipoprotein B-100: A Review. J. Clin. Lipidol..

[85] Tiscia G.L., Margaglione M. (2018). Human Fibrinogen: Molecular and Genetic Aspects of Congenital Disorders. Int. J. Mol. Sci..

[86] Blanco-Abad V., Noia M., Valle A., Fontenla F., Folgueira I., De Felipe A.P., Pereiro P., Leiro J., Lamas J. (2018). The Coagulation System Helps Control Infection Caused by the Ciliate Parasite *Philasterides dicentrarchi* in the Turbot *Scophthalmus maximus* (L.). Dev. Comp. Immunol..

[87] Kiriake A., Ohta A., Suga E., Matsumoto T., Ishizaki S., Nagashima Y. (2016). Comparison of Tetrodotoxin Uptake and Gene Expression in the Liver between Juvenile and Adult Tiger Pufferfish, Takifugu Rubripes. Toxicon.

[88] Weisel J.W., Litvinov R.I. (2013). Mechanisms of Fibrin Polymerization and Clinical Implications. Blood.

[89] Muller S., Radic M. (2015). Citrullinated Autoantigens: From Diagnostic Markers to Pathogenetic Mechanisms. Clin. Rev. Allergy Immunol..

[90] Blachère N.E., Parveen S., Frank M.O., Dill B.D., Molina H., Orange D.E. (2017). High-Titer Rheumatoid Arthritis Antibodies Preferentially Bind Fibrinogen Citrullinated by Peptidylarginine Deiminase 4. Arthritis Rheumatol..

[91] Hofman Z.L.M., De Maat S., Maas C. (2018). High-Molecular-Weight Kininogen: Breaking Bad in Lethal Endotoxemia. J. Thromb. Haemost..

[92] Al Hariri M., Elmedawar M., Zhu R., Jaffa M.A., Zhao J., Mirzaei P., Ahmed A., Kobeissy F., Ziyadeh F.N., Mechref Y. (2017). Proteome Profiling in the Aorta and Kidney of Type 1 Diabetic Rats. PLoS ONE.

[93] Armstrong P.B., Quigley J.P. (1999). Alpha2-Macroglobulin: An Evolutionarily Conserved Arm of the Innate Immune System. Dev. Comp. Immunol..

[94] Davies S.G., Sim R.B. (1981). Intramolecular General Acid Catalysis in the Binding Reactions of Alpha 2-Macroglobulin and Complement Components C3 and C4. Biosci. Rep..

[95] Sottrup-Jensen L., Stepanik T.M., Kristensen T., Lønblad P.B., Jones C.M., Wierzbicki D.M., Magnusson S., Domdey H., Wetsel R.A., Lundwall A. (1985). Common Evolutionary Origin of Alpha 2-Macroglobulin and Complement Components C3 and C4. Proc. Natl. Acad. Sci. USA.

[96] Dodds A.W., Law S.K. (1998). The Phylogeny and Evolution of the Thioester Bond-Containing Proteins C3, C4 and Alpha 2-Macroglobulin. Immunol. Rev..

[97] Fishelson Z., Attali G., Mevorach D. (2001). Complement and Apoptosis. Mol. Immunol..

[98] Dodds A.W. (2002). Which Came First, the Lectin/Classical Pathway or the Alternative Pathway of Complement?. Immunobiology.

[99] Lange S., Dodds A.W., Gudmundsdóttir S., Bambir S.H., Magnadottir B. (2005). The Ontogenic Transcription of Complement Component C3 and Apolipoprotein A-I tRNA in Atlantic Cod (*Gadus morhua* L.)—A Role in Development and Homeostasis?. Dev. Comp. Immunol..

[100] Lange S., Bambir S.H., Dodds A.W., Bowden T., Bricknell I., Espelid S., Magnadottir B. (2006). Complement Component C3 Transcription in Atlantic Halibut (*Hippoglossus hippoglossus* L.) Larvae. Fish Shellfish Immunol..

[101] Boshra H., Li J., Sunyer J.O. (2006). Recent Advances on the Complement System of Teleost Fish. Fish Shellfish Immunol..

[102] Nakao M., Tsujikura M., Ichiki S., Vo T.K., Somamoto T. (2011). The Complement System in Teleost Fish: Progress of Post-Homolog-Hunting Researches. Dev. Comp. Immunol..

[103] Hutchinson D., Clarke A., Heesom K., Murphy D., Eggleton P. (2017). Carbamylation/Citrullination of IgG Fc in Bronchiectasis, Established RA with Bronchiectasis and RA Smokers: A Potential Risk Factor for Disease. ERJ Open Res..

[104] Lundqvist M.L., Middleton D.L., Radford C., Warr G.W., Magor K.E. (2006). Immunoglobulins of the Non-Galliform Birds: Antibody Expression and Repertoire in the Duck. Dev. Comp. Immunol..

[105] de los Rios M., Criscitiello M.F., Smider V.V. (2015). Structural and Genetic Diversity in Antibody Repertoires from Diverse Species. Curr. Opin. Struct. Biol..

[106] Akula S., Hellman L. (2017). The Appearance and Diversification of Receptors for IgM during Vertebrate Evolution. Curr. Top. Microbiol. Immunol..

[107] Zhang X., Calvert R.A., Sutton B.J., Doré K. (2017). IgY: A Key Isotype in Antibody Evolution. Biol. Rev. Camb. Philos. Soc..

[108] Hellman N.E., Gitlin J.D. (2002). Ceruloplasmin Metabolism and Function. Annu. Rev. Nutr..

[109] Das S., Sahoo P.K. (2018). Ceruloplasmin, a Moonlighting Protein in Fish. Fish Shellfish Immunol..

[110] Lee K.A., Goetting V.S., Tell L.A. (2015). Inflammatory Markers Associated with Trauma and Infection in Red-Tailed Hawks (Buteo Jamaicensis) in the USA. J. Wildl. Dis..

[111] Pankov R., Yamada K.M. (2002). Fibronectin at a Glance. J. Cell Sci..

[112] Sato Y., Nagatoshi K., Hamano A., Imamura Y., Huss D., Uchida S., Lansford R. (2017). Basal Filopodia and Vascular Mechanical Stress Organize Fibronectin into Pillars Bridging the Mesoderm-Endoderm Gap. Development.

[113] Rick J.W., Chandra A., Dalle Ore C., Nguyen A.T., Yagnik G., Aghi M.K. (2019). Fibronectin in Malignancy: Cancer-Specific Alterations, Protumoral Effects, and Therapeutic Implications. Semin. Oncol..

[114] Kimura E., Kanzaki T., Tahara K., Hayashi H., Hashimoto S., Suzuki A., Yamada R., Yamamoto K., Sawada T. (2014). Identification of Citrullinated Cellular Fibronectin in Synovial Fluid from Patients with Rheumatoid Arthritis. Mod. Rheumatol..

[115] Stefanelli V.L., Choudhury S., Hu P., Liu Y., Schwenzer A., Yeh C.R., Chambers D.M., Pesson K., Li W., Segura T. (2019). Citrullination of Fibronectin Alters Integrin Clustering and Focal Adhesion Stability Promoting Stromal Cell Invasion. Matrix Biol..

[116] Giansanti F., Leboffe L., Pitari G., Ippoliti R., Antonini G. (2012). Physiological Roles of Ovotransferrin. Biochim. Biophys. Acta.

[117] Lambert L.A. (2012). Molecular Evolution of the Transferrin Family and Associated Receptors. Biochim. Biophys. Acta.

[118] Kushner I., Mackiewicz A. (1993). The Acute Phase Response: An Overview. Acute-Phase Glycoproteins: Molecular Biology, Biochemistry and Clinical Applications.

[119] Gettins P.G. (2002). Serpin Structure, Mechanism, and Function. Chem. Rev..

[120] Guttman O., Baranovski B.M., Schuster R., Kaner Z., Freixo-Lima G.S., Bahar N., Mizrahi M.I., Brami I., Ochayon D.E., Lewis E.C. (2015). Acute-Phase Protein α1-Anti-Trypsin: Diverting Injurious Innate and Adaptive Immune Responses from Non-Authentic Threats. Clin. Exp. Immunol..

[121] Mostert V. (2000). Selenoprotein P: Properties, Functions, and Regulation. Arch. Biochem. Biophys..

[122] Kolarich D., Weber A., Turecek P.L., Schwarz H.P., Altmann F. (2006). Comprehensive Glyco-Proteomic Analysis of Human Alpha1-Antitrypsin and Its Charge Isoforms. Proteomics.

[123] Burk R.F., Hill K.E. (2009). Selenoprotein P-Expression, Functions, and Roles in Mammals. Biochim. Biophys. Acta.

[124] Cao N., Li W., Li B., Tian Y., Xu D. (2017). Transcriptome Profiling Reveals the Immune Response of Goose T Cells under Selenium Stimuli. Anim. Sci. J..

[125] Huang J.Q., Ren F.Z., Jiang Y.Y., Xiao C., Lei X.G. (2015). Selenoproteins Protect Against Avian Nutritional Muscular Dystrophy by Metabolizing Peroxides and Regulating Redox/Apoptotic Signaling. Free Radic. Biol. Med..

[126] Wang Y.X., Xiao X., Zhan X.A. (2018). Antagonistic Effects of Different Selenium Sources on Growth Inhibition, Oxidative Damage, and Apoptosis Induced by Fluorine in Broilers. Poult. Sci..

[127] Lobanov A.V., Hatfield D.L., Gladyshev V.N. (2008). Reduced Reliance on the Trace Element Selenium during Evolution of Mammals. Genome Biol..

[128] Tamburrini M., Riccio A., Romano M., Giardina B., di Prisco G. (2000). Structural and Functional Analysis of the Two Haemoglobins of the Antarctic Seabird *Catharacta maccormicki* Characterization of an additional Phosphate Binding Site by Molecular Modelling. Eur. J. Biochem..

[129] Riccio A., Tamburrini M., Giardina B., di Prisco G. (2001). Molecular Dynamics Analysis of a Second Phosphate Site in the Hemoglobins of the Seabird, South Polar Skua. Is there a Site-Site Migratory Mechanism along the Central Cavity?. Biophys. J..

[130] Bikle D.D., Schwartz J. (2019). Vitamin D Binding Protein, Total and Free Vitamin D Levels in Different Physiological and Pathophysiological Conditions. Front. Endocrinol..

[131] Verboven C., Rabijns A., De Maeyer M., Van Baelen H., Bouillon R., De Ranter C. (2002). A Structural Basis for the Unique Binding Features of the Human Vitamin D-Binding Protein. Nat. Struct. Biol..

[132] Yen C.F., Lin E.C., Wang Y.H., Wang P.H., Lin H.W., Hsu J.C., Wu L.S., Jiang Y.N., Ding S.T. (2009). Abundantly Expressed Hepatic Genes and Their Differential Expression in Liver of Prelaying and Laying Geese. Poult. Sci..

[133] Yamamoto N., Suyama H., Yamamoto N. (2008). Immunotherapy for Prostate Cancer with Gc Protein-Derived Macrophage-Activating Factor, GcMAF. Transl. Oncol..

[134] Tarighi S., Najafi M., Hossein-Nezhad A., Ghaedi H., Meshkani R., Moradi N., Fadaei R., Kazerouni F., Shanaki M. (2017). Association Between Two Common Polymorphisms of Vitamin D Binding Protein and the Risk of Coronary Artery Disease: A Case-Control Study. J. Med. Biochem..

[135] Kilpatrick L.E., Phinney K.W. (2017). Quantification of Total Vitamin-D-Binding Protein and the Glycosylated Isoforms by Liquid Chromatography-Isotope Dilution Mass Spectrometry. J. Proteome Res..

[136] Leavesley D.I., Kashyap A.S., Croll T., Sivaramakrishnan M., Shokoohmand A., Hollier B.G., Upton Z. (2013). Vitronectin-Master Controller or Micromanager?. IUBMB Life.

[137] Felding-Habermann B., Cheresh D.A. (1993). Vitronectin and Its Receptors. Curr. Opin. Cell Biol..

[138] Mikrou A., Zarkadis I.K. (2010). Cloning of the Sixth Complement Component and, Spatial and Temporal Expression Profile of MAC Structural and Regulatory Genes in Chicken. Dev. Comp. Immunol..

[139] Preissner K.T., Seiffert D. (1998). Role of Vitronectin and its Receptors in Haemostasis and Vascular Remodeling. Thrombosis. Res..

[140] Hurt E.M., Chan K., Serrat M.A.D., Thomas S.B., Veenstra T.D., Farrar W.L. (2010). Identification of Vitronectin as an Extrinsic Inducer of Cancer Stem Cell Differentiation and Tumor Formation. Stem. Cells.

[141] Rice H.C., Townsend M., Bai J., Suth S., Cavanaugh W., Selkoe D.J., Young-Pearse T.L. (2012). Pancortins Interact with Amyloid Precursor Protein and Modulate Cortical Cell Migration. Development.

[142] Pronker M.F., Bos T.G., Sharp T.H., Thies-Weesie D.M., Janssen B.J. (2015). Olfactomedin-1 Has a V-shaped Disulfide-Linked Tetrameric Structure. J. Biol. Chem..

[143] Pandya N.J., Seeger C., Babai N., Gonzalez-Lozano M.A., Mack V., Lodder J.C., Gouwenberg Y., Mansvelder H.D., Danielson U.H., Li K.W. (2018). Noelin1 Affects Lateral Mobility of Synaptic AMPA Receptors. Cell Rep..

[144] Shi W., Ye Z., Zhuang L., Li Y., Shuai W., Zuo Z., Mao X., Liu R., Wu J., Chen S. (2016). Olfactomedin 1 Negatively Regulates NF-κB Signalling and Suppresses the Growth and Metastasis of Colorectal Cancer Cells. J. Pathol..

[145] Lencinas A., Chhun D.C., Dan K.P., Ross K.D., Hoover E.A., Antin P.B., Runyan R.B. (2013). Olfactomedin-1 Activity Identifies a Cell Invasion Checkpoint during Epithelial-Mesenchymal Transition in the Chick Embryonic Heart. Dis. Model Mech..

[146] Wakabayashi S. (2013). New Insights into the Functions of Histidine-Rich Glycoprotein. Int. Rev. Cell Mol. Biol..

[147] Jones A.L., Hulett M.D., Parish C.R. (2005). Histidine-Rich Glycoprotein: A Novel Adaptor Protein in Plasma that Modulates the Immune, Vascular and Coagulation Systems. Immunol. Cell Biol..

[148] Blank M., Shoenfeld Y. (2008). Histidine-Rich Glycoprotein Modulation of Immune/Autoimmune, Vascular, and Coagulation Systems. Clin. Rev. Allergy Immunol..

[149] Poon I.K., Patel K.K., Davis D.S., Parish C.R., Hulett M.D. (2011). Histidine-Rich Glycoprotein: The Swiss Army Knife of Mammalian Plasma. Blood.

[150] Johnson L.D., Goubran H.A., Kotb R.R. (2014). Histidine Rich Glycoprotein and Cancer: A Multi-Faceted Relationship. Anticancer Res..

[151] Wisniewska M., Happonen L., Kahn F., Varjosalo M., Malmström L., Rosenberger G., Karlsson C., Cazzamali G., Pozdnyakova I., Frick I.M. (2014). Functional and Structural Properties of a Novel Protein and Virulence Factor (Protein sHIP) in *Streptococcus pyogenes*. J. Biol. Chem..

[152] Jaken S., Parker P.J. (2000). Protein Kinase C Binding Partners. Bioessays.

[153] Matsuhashi S., Noji S., Koyama E., Myokai F., Ohuchi H., Taniguchi S., Hori K. (1996). New Gene, Nel, Encoding a Mr 91 K Protein with EGF-Like Repeats is Strongly Expressed in Neural Tissues of Early Stage Chick Embryos. Dev. Dyn..

[154] Nakamura R., Nakamoto C., Obama H., Durward E., Nakamoto M. (2012). Structure-Function Analysis of Nel, a Thrombospondin-1-Like Glycoprotein Involved in Neural Development and Functions. J. Biol. Chem..

[155] Silverman G.A., Bird P.I., Carrell R.W., Church F.C., Coughlin P.B., Gettins P.G., Irving J.A., Lomas D.A., Luke C.J., Moyer R.W. (2001). The Serpins are an Expanding Superfamily of Structurally Similar but Functionally Diverse proteins. Evolution, Mechanism of Inhibition, Novel Functions, and a Revised Nomenclature. J. Biol. Chem..

[156] Law R.H., Zhang Q., McGowan S., Buckle A.M., Silverman G.A., Wong W., Rosado C.J., Langendorf C.G., Pike R.N., Bird P.I. (2006). An Overview of the Serpin Superfamily. Genome Biol..

[157] Whisstock J.C., Bottomley S.P. (2006). Molecular Gymnastics: Serpin Structure, Folding and Misfolding. Curr. Opin. Struct. Biol..

[158] Njålsson R., Norgren S. (2005). Physiological and Pathological Aspects of GSH Metabolism. Acta Paediatr..

[159] Nitto T., Inoue T., Node K. (2008). Alternative Spliced Variants in the Pantetheinase Family of Genes Expressed in Human Neutrophils. Gene.

[160] Bartucci R., Salvati A., Olinga P., Boersma Y.L. (2019). Vanin 1: Its Physiological Function and Role in Diseases. Int. J. Mol. Sci..

[161] Naquet P., Pitari G., Duprè S., Galland F. (2014). Role of the Vnn1 Pantetheinase in Tissue Tolerance to Stress. Biochem. Soc. Trans..

[162] Martin F., Malergue F., Pitari G., Philippe J.M., Philips S., Chabret C., Granjeaud S., Mattei M.G., Mungall A.J., Naquet P. (2001). Vanin Genes are Clustered (Human 6q22-24 and Mouse 10A2B1) and Encode Isoforms of Pantetheinase Ectoenzymes. Immunogenetics.

[163] Nitto T., Onodera K. (2013). Linkage between Coenzyme a Metabolism and Inflammation: Roles of Pantetheinase. J. Pharmacol. Sci..

[164] Jansen P.A., Kamsteeg M., Rodijk-Olthuis D., van Vlijmen-Willems I.M., de Jongh G.J., Bergers M., Tjabringa G.S., Zeeuwen P.L., Schalkwijk J. (2009). Expression of the Vanin Gene Family in Normal and Inflamed Human Skin: Induction by Proinflammatory Cytokines. J. Investig. Dermatol..

[165] Wang N., Qin X., Cao Y., Liang B., Yu K., Ye H. (2018). Plasma Vascular Non-Inflammatory Molecule 3 is Associated with Gastrointestinal Acute Graft-Versus-Host Disease in Mice. J. Inflamm..

[166] McDonnell T., Artim-Esen B., Wincup C., Ripoll V.M., Isenberg D., Giles I.P., Rahman A., Pericleous C. (2018). Antiphospholipid Antibodies to Domain I of Beta-2-Glycoprotein I Show Different Subclass Predominance in Comparison to Antibodies to Whole Beta-2-glycoprotein I. Front. Immunol..

[167] El-Assaad F., Krilis S.A., Giannakopoulos B. (2016). Posttranslational Forms of beta 2-Glycoprotein I in the Pathogenesis of the Antiphospholipid Syndrome. Thromb. J..

[168] El-Assaad F., Qi M., Gordon A.K., Qi J., Dong S., Passam F., Weaver J.C., Giannakopoulos B., Krilis S.A. (2017). Beta 2-Glycoprotein I Protects Mice Against Gram-Negative Septicaemia in a Sexually Dimorphic Manner. Sci. Rep..

[169] Zhou S., Chen G., Qi M., El-Assaad F., Wang Y., Dong S., Chen L., Yu D., Weaver J.C., Beretov J. (2016). Gram Negative Bacterial Inflammation Ameliorated by the Plasma Protein Beta 2-Glycoprotein I. Sci. Rep..

[170] McDonnell T., Wincup C., Buchholz I., Pericleous C., Giles I., Ripoll V., Cohen H., Delcea M., Rahman A. (2019). The Role of Beta-2-Glycoprotein I in Health and Disease Associating Structure with Function: More than just APS. Blood Rev..

[171] Shi X., Ohta Y., Liu X., Shang J., Morihara R., Nakano Y., Feng T., Huang Y., Sato K., Takemoto M. (2019). Acute Anti-Inflammatory Markers ITIH4 and AHSG in Mice Brain of a Novel Alzheimer’s Disease Model. J. Alzheimers Dis..

[172] Zhuo L., Hascall V.C., Kimata K. (2004). Inter-Alpha-Trypsin Inhibitor, a Covalent Protein-Glycosaminoglycan-Protein Complex. J. Biol. Chem..

[173] Barrios-Anderson A., Chen X., Nakada S., Chen R., Lim Y.P., Stonestreet B.S. (2019). Inter-Alpha Inhibitor Proteins Modulate Neuroinflammatory Biomarkers after Hypoxia-Ischemia in Neonatal Rats. J. Neuropathol. Exp. Neurol..

[174] Stober V.P., Lim Y.P., Opal S., Zhuo L., Kimata K., Garantziotis S. (2019). Inter-α-Inhibitor Ameliorates Endothelial Inflammation in Sepsis. Lung.

[175] Htwe S.S., Wake H., Liu K., Teshigawara K., Stonestreet B.S., Lim Y.P., Nishibori M. (2018). Inter-α Inhibitor Proteins Maintain Neutrophils in a Resting State by Regulating Shape and Reducing ROS Production. Blood Adv..

[176] Sondheimer N., Fang J.K., Polyak E., Falk M.J., Avadhani N.G. (2010). Leucine-Rich Pentatricopeptide-Repeat Containing Protein Regulates Mitochondrial Transcription. Biochemistry.

[177] Martínez-Godínez M.A., Cruz-Domínguez M.P., Jara L.J., Domínguez-López A., Jarillo-Luna R.A., Vera-Lastra O., Montes-Cortes D.H., Campos-Rodríguez R., López-Sánchez D.M., Mejía-Barradas C.M. (2015). Expression of NLRP3 Inflammasome, Cytokines and Vascular Mediators in the Skin of Systemic Sclerosis Patients. Isr. Med. Assoc. J..

[178] Kang W., Reid K.B. (2003). DMBT1, a Regulator of Mucosal Homeostasis through the Linking of Mucosal Defense and Regeneration?. FEBS Lett..

[179] Ligtenberg A.J., Karlsson N.G., Veerman E.C. (2010). Deleted in Malignant Brain Tumors-1 Protein (DMBT1): A Pattern Recognition Receptor with Multiple Binding Sites. Int. J. Mol. Sci..

[180] Li J., Metruccio M.M.E., Evans D.J., Fleiszig S.M.J. (2017). Mucosal Fluid Glycoprotein DMBT1 Suppresses Twitching Motility and Virulence of the Opportunistic Pathogen Pseudomonas aeruginosa. PLoS Pathog..

[181] Deng H., Gao Y.B., Wang H.F., Jin X.L., Xiao J.C. (2012). Expression of Deleted in Malignant Brain Tumours 1 (DMBT1) Relates to the Proliferation and Malignant Transformation of Hepatic Progenitor Cells in Hepatitis B Virus-Related Liver Diseases. Histopathology.

[182] Rosenstiel P., Sina C., End C., Renner M., Lyer S., Till A., Hellmig S., Nikolaus S., Fölsch U.R., Helmke B. (2007). Regulation of DMBT1 via NOD2 and TLR4 in Intestinal Epithelial Cells Modulates Bacterial Recognition and Invasion. J. Immunol..

[183] Mollenhauer J., Wiemann S., Scheurlen W., Korn B., Hayashi Y., Wilgenbus K.K., von Deimling A., Poustka A. (1997). DMBT1, a New Member of the SRCR Superfamily, on Chromosome 10q25.3-26.1 is Deleted in Malignant Brain Tumours. Nat. Genet..

[184] Mori M., Shiraishi T., Tanaka S., Yamagata M., Mafune K., Tanaka Y., Ueo H., Barnard G.F., Sugimachi K. (1999). Lack of DMBT1 Expression in Oesophageal, Gastric and Colon Cancers. Br. J. Cancer.

[185] Mollenhauer J., Herbertz S., Helmke B., Kollender G., Krebs I., Madsen J., Holmskov U., Sorger K., Schmitt L., Wiemann S. (2001). Deleted in Malignant Brain Tumors 1 is a Versatile Mucin-Like Molecule Likely to Play a Differential Role in Digestive Tract Cancer. Cancer Res..

[186] Robbe C., Paraskeva C., Mollenhauer J., Michalski J.C., Sergi C., Corfield A. (2005). DMBT1 Expression and Glycosylation during the Adenoma-Carcinoma Sequence in Colorectal Cancer. Biochem. Soc. Trans..

[187] Tuttolomondo M., Casella C., Hansen P.L., Polo E., Herda L.M., Dawson K.A., Ditzel H.J., Mollenhauer J. (2017). Human DMBT1-Derived Cell-Penetrating Peptides for Intracellular siRNA Delivery. Mol. Ther. Nucleic Acids.

[188] Sarrias M.R., Grønlund J., Padilla O., Madsen J., Holmskov U., Lozano F. (2004). The Scavenger Receptor Cysteine-Rich (SRCR) Domain: An Ancient and Highly Conserved Protein Module of the Innate Immune System. Crit. Rev. Immunol..

[189] Bessa Pereira C., Bocková M., Santos R.F., Santos A.M., Martins de Araújo M., Oliveira L., Homola J., Carmo A.M. (2016). The Scavenger Receptor SSc5D Physically Interacts with Bacteria through the SRCR-Containing N-Terminal Domain. Front. Immunol..

[190] Balakrishnan L., Bhattacharjee M., Ahmad S., Nirujogi R.S., Renuse S., Subbannayya Y., Marimuthu A., Srikanth S.M., Raju R., Dhillon M. (2014). Differential Proteomic Analysis of Synovial Fluid from Rheumatoid Arthritis and Osteoarthritis Patients. Clin. Proteom..

[191] Meyer E.J., Nenke M.A., Rankin W., Lewis J.G., Torpy D.J. (2016). Corticosteroid-Binding Globulin: A Review of Basic and Clinical Advances. Horm. Metab. Res..

[192] Bae Y.J., Kratzsch J. (2015). Corticosteroid-Binding Globulin: Modulating Mechanisms of Bioavailability of Cortisol and Its Clinical Implications. Best Pract. Res. Clin. Endocrinol. Metab..

[193] Lattin C.R., Breuner C.W., Michael Romero L. (2016). Does Corticosterone Regulate the Onset of Breeding in Free-living Birds? The CORT-Flexibility Hypothesis and Six Potential Mechanisms for Priming Corticosteroid Function. Horm. Behav..

[194] Rensel M.A., Schlinger B.A. (2016). Determinants and Significance of Corticosterone Regulation in the Songbird Brain. Gen. Comp. Endocrinol..

[195] Quadro L., Hamberger L., Colantuoni V., Gottesman M.E., Blaner W.S. (2003). Understanding the Physiological Role of Retinol-Binding Protein in Vitamin A Metabolism Using Transgenic and Knockout Mouse Models. Mol. Asp. Med..

[196] Yang Q., Graham T.E., Mody N., Preitner F., Peroni O.D., Zabolotny J.M., Kotani K., Quadro L., Kahn B.B. (2005). Serum Retinol Binding Protein 4 Contributes to Insulin Resistance in Obesity and Type 2 Diabetes. Nature.

[197] Moraes-Vieira P.M., Yore M.M., Dwyer P.M., Syed I., Aryal P., Kahn B.B. (2014). RBP4 Activates Antigen-Presenting Cells, Leading to Adipose Tissue Inflammation and Systemic Insulin Resistance. Cell Metab..

[198] Herman M.A., Kahn B.B. (2006). Glucose Transport and Sensing in the Maintenance of Glucose Homeostasis and Metabolic Harmony. J. Clin. Investig..

[199] Jaconi S., Rose K., Hughes G.J., Saurat J.H., Siegenthaler G. (1995). Characterization of Two Post-Translationally Processed Forms of Human Serum Retinol-Binding Protein: Altered Ratios in Chronic Renal Failure. J. Lipid Res..

[200] Fang Y., Shen X. (2017). Ubiquitin Carboxyl-Terminal Hydrolases: Involvement in Cancer Progression and Clinical Implications. Cancer Metastasis Rev..

[201] Bishop P., Rocca D., Henley J.M. (2016). Ubiquitin C-Terminal Hydrolase L1 (UCH-L1): Structure, Distribution and Roles in Brain Function and Dysfunction. Biochem. J..

[202] Thelin E., Al Nimer F., Frostell A., Zetterberg H., Blennow K., Nyström H., Svensson M., Bellander B.M., Piehl F., Nelson D.W. (2019). A Serum Protein Biomarker Panel Improves Outcome Prediction in Human Traumatic Brain Injury. J. Neurotrauma.

[203] Tian L., Wang K., Liu H., Li K., Lin B., Fang Z., Han J., Li N., Yang H., Bian L. (2019). UCH-L1 Mitigates Neurotoxicity Induced by ZnO Particles via Stabilizing the Inhibitor of NF-Kappa B Signaling, IκB-α. Ecotoxicol. Environ. Saf..

[204] Matuszczak E., Tylicka M., Dębek W., Sankiewicz A., Gorodkiewicz E., Hermanowicz A. (2017). Overexpression of Ubiquitin Carboxyl-Terminal Hydrolase L1 (UCHL1) in Serum of Children after Thermal Injury. Adv. Med. Sci..

[205] Woo S.K., Baek S.H., Lee J.I., Yoo Y.J., Cho C.M., Kang M.S., Chung C.H. (1997). Purification and Characterization of a New Ubiquitin C-Terminal Hydrolase (UCH-1) with Isopeptidase Activity from Chick Skeletal Muscle. J. Biochem..

[206] Vigier S., Gagnon H., Bourgade K., Klarskov K., Fülöp T., Vermette P. (2017). Composition and Organization of the Pancreatic Extracellular Matrix by Combined Methods of Immunohistochemistry, Proteomics and Scanning Electron Microscopy. Curr. Res. Transl. Med..

[207] Van den Berg T.K., van der Ende M., Döpp E.A., Kraal G., Dijkstra C.D. (1993). Localization of Beta 1 Integrins and Their Extracellular Ligands in Human Lymphoid Tissues. Am. J. Pathol..

[208] Cai Y., Beziau A., Sich M., Kleppel M.M., Gubler M.C. (1996). Collagen Distribution in Human Membranous Glomerulonephritis. Pediatr. Nephrol..

[209] Moriggi M., Pastorelli L., Torretta E., Tontini G.E., Capitanio D., Bogetto S.F., Vecchi M., Gelfi C. (2017). Contribution of Extracellular Matrix and Signal Mechanotransduction to Epithelial Cell Damage in Inflammatory Bowel Disease Patients: A Proteomic Study. Proteomics.

[210] Schaeffer J., Tannahill D., Cioni J.M., Rowlands D., Keynes R. (2018). Identification of the Extracellular Matrix Protein Fibulin-2 as a Regulator of Spinal Nerve Organization. Dev. Biol..

[211] Calpena E., Palau F., Espinós C., Galindo M.I. (2015). Evolutionary History of the Smyd Gene Family in Metazoans: A Framework to Identify the Orthologs of Human Smyd Genes in Drosophila and Other Animal Species. PLoS ONE.

[212] Du S.J., Tan X., Zhang J. (2014). SMYD Proteins: Key Regulators in Skeletal and Cardiac Muscle Development and Function. Anat. Rec..

[213] Tracy C., Warren J.S., Szulik M., Wang L., Garcia J., Makaju A., Russell K., Miller M., Franklin S. (2018). The Smyd Family of Methyltransferases: Role in Cardiac and Skeletal Muscle Physiology and Pathology. Curr. Opin. Physiol..

[214] Song J., Liu Y., Chen Q., Yang J., Jiang Z., Zhang H., Liu Z., Jin B. (2019). Expression Patterns and the Prognostic Value of the SMYD Family Members in Human Breast Carcinoma Using Integrative Bioinformatics Analysis. Oncol. Lett..

